# Seminal plasma amino acid profile in different breeds of chicken: Role of seminal plasma on sperm cryoresistance

**DOI:** 10.1371/journal.pone.0209910

**Published:** 2019-01-04

**Authors:** Julián Santiago-Moreno, Berenice Bernal, Serafín Pérez-Cerezales, Cristina Castaño, Adolfo Toledano-Díaz, Milagros C. Esteso, Alfonso Gutiérrez-Adán, Antonio López-Sebastián, María G. Gil, Henri Woelders, Elisabeth Blesbois

**Affiliations:** 1 Departamento de Reproducción Animal, INIA, Madrid, Spain; 2 Departamento de Mejora Genética Animal, INIA, Madrid, Spain; 3 Wageningen University and Research, Animal Breeding and Genomics, Wageningen, the Netherlands; 4 UMR Physiologie de la Reproduction et des Comportements, INRA-CNRS-Université François Rabelais-Haras Nationaux, Nouzilly, France; Universidad Autonoma de Queretaro, MEXICO

## Abstract

Seminal plasma is a key biological fluid that modulates sperm function in the reproduction process. However, its role in sperm biotechnologies is scarce in poultry. The aims of the present study were to study the amino acids profile and total proteins of seminal plasma in 12 Spanish chicken breeds and to investigate the role of seminal plasma on cryoresistance of rooster sperm. To investigate the role of seminal plasma on cryoresistance, diluted pooled semen samples were cryopreserved in the presence and absence of seminal plasma. Glutamic acid was the most abundant free amino acid in seminal plasma, followed by alanine, serine, valine, and glycine. There was an influence of breed (P<0.05) on the percentage of viable sperm after freezing-thawing of samples with seminal plasma. Cluster analysis revealed that White Prat, Black Castellana, Blue Andaluza, Quail Castellana, and Red-Barred Vasca returned the best freezing-thawing response (good freezers). There was a positive correlation between seminal plasma concentrations of valine, isoleucine lysine, leucine and post thaw viability. The evaluation of fertilization capacity of frozen-thawed semen from the breeds White Prat (‘good freezer’) and Black-Red Andaluza (‘bad freezer’) showed that good freezer had higher fertility (20/68, 29.4%) compared to bad freezer breed (14/76, 18.4%), even if the difference was not significant (P = 0.08). The TUNEL assay revealed that freezing/thawing procedures in presence of seminal plasma provoked higher DNA fragmentation in most of the breeds, with a positive correlation between seminal alanine, valine, isoleucine, methionine, leucine, tyrosine, phenylalanine concentrations and DNA integrity. DNA fragmentation was lower in absence of seminal plasma and the breed effect on sperm viability was highly reduced. It is concluded that specific seminal plasma amino acids were associated with post-thaw percentage of viable sperm and DNA integrity. The removal of seminal plasma decreases the variability of the results and DNA fragmentation damages.

## Introduction

The influence of seminal plasma on sperm storage may vary among species. Its removal is recommended in the majority of semen cryopreservation protocols of species such as caprine in order to ensure maximal sperm viability [1], but it isn’t entirely recommended in other mammals (e.g. ovine; [2]). Mammalian seminal plasma may contain factors that influence resistance of sperm to cold-shock damage and may prevent cryoinjury [3–5]. Conversely, detrimental effects of seminal plasma on sperm variables after freezing have also been reported [1,6,7]. In birds, early preliminary studies showed contrasted effects of seminal plasma fractions on refrigerated rooster sperm [8], and a global deleterious effect in chickens and turkeys [9,10], but its effects on frozen semen have never been studied. Rooster semen is usually frozen complete, i.e with presence of seminal plasma. However, the effects (advantages or disadvantages) of seminal plasma during cryopreservation of rooster semen are not clear. Seminal plasma provides metabolic support, as energy sources for the sperm cells, and influences sperm functionality in a not completely understood way.

The components of rooster seminal plasma derive from the proximal efferent ducts, epididymides and deferent ducts [11]. During natural mating, the transparent fluid from the paracloacal vascular bodies joins to the deferent duct fluid [12]. Besides inorganics ions (Na^+^, K^+^, Ca^+^) [13], a characteristic biochemical feature of the seminal plasma is the occurrence of a wide range of organic constituents such as carbohydrates, lipids, lipoprotein complexes, proteins, peptides, and amino acids [8,14]. The functional significance of free amino acids is diverse: scavenge free radicals, act as a solute protecting the cell against the denaturing effects of hyperosmolality, provide buffers with protective influence on sperm cells, and serve as oxidizable substrates for spermatozoa, [15]. The role of seminal plasma amino acids during cryopreservation process is not clear, but it has been described that a number of amino acids have a cryoprotective effect during freezing and thawing of mammalian sperm [16,17], or of isolated enzymes, such as calcium ATPase [18] or phosphofructokinase [19]. Moreover, some organisms accumulate amino acids in response to cold temperatures [20]. Several studies have focused on their use as additives in extenders. Glutamine, proline, histidine, glycine, alanine have been used for cryopreservation of ram, stallion, goat and human semen [21–24]. Although the cryoprotectant mechanism of amino acids is not well known, some hypotheses regarding possible mechanisms have been provided, such as anti-oxidative activity [25] and protection against denaturing effects of low water potential during freezing [26]. Carpenter and Crowe [19] noted that amino acid might stabilize proteins, thus avoiding denaturation and dissociation that would lead to a greater contact surface between proteins and solutes during freeze-thaw process.

The seminal plasma amino acid profile may vary among genotypes [11], and differences between chicken breeds may be expected. Individual semen donors, or breeds may also differ in ‘freezability’ of the semen, and be categorized as ‘‘good” or ‘‘bad freezers”. The mechanisms underlying differences in cryosensitivity between different individuals have yet to be elucidated. It has been demonstrated that consistent inter-individual variations in sperm freezability are genetically determined [27]. Considering the above mentioned cryoprotective properties of amino acids, we may suggest that a possible variability among chicken breeds to sustain sperm cryopreservation could be related to differences in seminal plasma amino acid compositions and protein content. Therefore, the aims of the present study were to investigate the role of seminal plasma and seminal amino acids profiles of different chicken breeds on sperm cryoresistance variability.

## Material and methods

### Experimental birds

The birds used in this study were of 12 Spanish chicken breeds (Black-Barred Andaluza, Black-Red Andaluza, Blue Andaluza, Black Castellana, Buff Prat, White Prat, Red-Barred Vasca, Red Villafranquina, Birchen Leonesa, White-Faced Spanish, Quail Castellana and Quail Silver Castellana). One hundred forty four roosters (12 of each breed), all of which were one year old at the beginning of the experiment were used for the collection of semen. In addition, hens of White Prat and Black-Red Andaluza breeds (30 hens per breed) were later used for insemination experiments. All animals were housed under natural photoperiod and temperature conditions in two 12 m^2^ sand-floor pens with partial roof cover at the El Encín Research Station (Madrid, Spain, 40° 31’ N). These birds were raised as part of the INIA’s genetic resources conservation program [28, 29]. All birds were fed a commercial feed containing 16% CP, 2700 kcal of ME/kg, 3.5% Ca and 0.5% available P over the entire experimental period. Animals were handled according to procedures approved by the INIA Ethics Committee (Órgano Regulador de los Comités de Ética de Experimentación Animal, reference number ORCEEA 2016/001) and were performed in accordance with the Spanish Policy for Animal Protection (RD53/2013), which conforms to European Union Directive 86/609 regarding the protection of animals used in scientific experiments.

### Experimental design

The amino acid profile and total proteins in seminal plasma were studied within each breed. A pool of seminal plasma for each breed was obtained every month, from August to November, and amino acid and total protein analysed in them (n = 48; 4 per breed). Mean sperm concentrations were also evaluated within each breed.

To investigate the role of seminal plasma on cryoresistance of rooster sperm, diluted pooled semen samples (n = 168; 14 replicates per breed x 12 breeds) collected from June to November, were divided into two aliquots. One aliquot was frozen with presence of seminal plasma, and the other one was frozen after removal of seminal plasma by centrifugation. Sperm variables were analysed before and after freezing-thawing.

The fertilization capacity of frozen semen from one ‘good freezer’ and one ‘bad freezer’ breed, as judged from the obtained in vitro post-thaw sperm assessments, was estimated in an artificial insemination (AI) experiment from the percentage fertile eggs resulting from two consecutive intravaginal AI, three days apart, of a total of 60 hens (30 per breed). Hens of each breed were used for testing fertilizing ability of frozen-thawed semen of the two mentioned breeds; i.e. 15 hens belonging to good freezer breed were inseminated with semen of good freezer, and the remaining 15 with semen of bad freezer; the same criterion was used for the hens of bad freezer breed.

### Semen collection, management and freezing

Semen was collected twice weekly over the study period, in 15-mL graduated centrifuge tubes (Sterilin) using the massage technique described by Burrows and Quinn [30]. Pools of semen for each breed were made on each occasion. Samples were managed differently, depending on whether they were used for seminal plasma amino acid analysis, or for freezing. Seminal plasma for amino acid assay was obtained by centrifugation of pooled raw semen at 1400g for 30 min. The plasma was evaluated by microscopy to ensure the absence of cells. If any cell were seen, a second centrifugation was made. The pellet was discarded. When semen samples were used in freezing experiments, each pool of semen was immediately diluted 1:1 (v/v) at field temperature using a Lake-Ravie medium [31] composed of sodium glutamate (1.92 g), glucose (0.8 g), magnesium acetate 4H_2_O (0.08 g), potassium acetate (0.5 g), polyvinylpyrrolidone (*M*_r_ 10 000; 0.3 g) and 100 mL H_2_O (final pH 7.08, final osmolality 343 mOsm/kg; hereinafter referred to as Lake and Ravie medium). This diluted, pooled semen was then immediately placed at 5°C, transported to the laboratory, and sperm concentration and sperm variables (sperm motility variables, plasma membrane integrity) examined (within 45 min of collection). Afterward, each pool was divided into two aliquots. One aliquot, diluted as required with Lake and Ravie medium to a concentration of 1200 × 10^6^ sperm/mL (aliquot with presence of seminal plasma). In the other one (aliquot without seminal plasma) the seminal plasma was removed by dilution with Lake-Centri diluent (1:4 v/v) and centrifugation at 600 g during 20 min prior to freezing. Briefly, the Lake-Centri medium was composed of 1000 ml H_2_O, 1.28 g potassium citrate tribasic monohydrate, 19.2 g sodium-L-glutamate, 6.0 g D-fructose, 5.0 g TES, 5.1 g sodium acetate trihydrate, 0.8 g magnesium acetate tetrahydrate, and 5.2 ml of 1N sodium hydroxide (340–350 mOsm/kg, pH = 7.0–7.2). The pellet obtained was reconstituted with Lake-Ravie medium. Both aliquot with and without seminal plasma were diluted with Lake-Ravie medium to a final concentration of 1200 × 10^6^ sperm/mL. Pure (≥99%) glycerol (GLY) was then added to the diluted samples, to leave a final 8% concentration (vol/vol), and equilibrated for 10 min at 5°C. After equilibration, the samples were loaded into 0.25 mL French straws and then frozen in two steps, i.e., from 5°C to −35°C at 7°C/min, and then from −35°C to −140°C at 60°C/min [32]. Freezing was performed using a Computer Freezer-Icetube 1810 freezer unit (Minitüb, Tiefenbach, Germany). The frozen straws were then plunged into and maintained in liquid nitrogen (at -196°C) until thawing. For thawing, the straws were warmed for 3 min in a water bath at 5°C.

### Amino acid and total protein assay

Seminal plasma obtained for amino acid assay (see above) was immediately stored at -20°C until determination of the seminal plasma free amino acid composition. Separation and determination of amino acid was made by ion exchange column chromatography [33,34]. Briefly, the samples were initially precipitated with three volumes of ice-cold acetone and incubated for 2h at—20°C. After centrifugation, the supernatant was removed and freeze dried in a speed vac. The pellet was redissolved in citrate buffer and applied to an ion exchange chromatography amino acid analyzer (Biochrom 30) using post column derivatization with ninhydrin. The ninhydrin reacts with amino acids forming a dye complex. Total protein was assessed by the Coomassie (Bradford) Protein Assay Kit (Thermo Scientific). The seminal plasma was diluted 10 times with Milli-Q water, then, 0.03 mL of the diluted plasma was mixed with 1.5 mL of the Coomassie reagent. The samples were incubated 10 min at room temperature and were analysed by measuring the absorbance at 595 nm (Agilent 8453 Spectrophotometer). The protein concentration was determined by a BSA standard curve with a linear working range of 25–500 μg/mL.

### Assessment of sperm variables

Sperm concentration and motility were assayed using a computer-aided sperm analyses (CASA) system coupled to a phase contrast microscope (Nikon Eclipse model 50i; Nikon Instruments Europe B.V., Izasa S.A.; negative contrast) and employing Sperm Class Analyzer (SCA, Barcelona, Spain) v.4.0. software (Microptic S.L., Barcelona, Spain) [35]. For motility analysis, sperm samples were diluted to a concentration of approximately 40 million sperm/ml and loaded onto warmed (38°C) 20 μm Leja 8-chamber slides (Leja Products B.V., Nieuw-Vennep, The Netherlands). The percentage of motile spermatozoa and the percentage showing progressive motility were recorded. Sperm movement characteristics—curvilinear velocity (VCL), straight-line velocity (VSL), average path velocity (VAP), amplitude of lateral head displacement (ALH), and beat-cross frequency (BCF)—were also recorded. Three progression ratios, expressed as percentages, were calculated from the velocity measurements described above: linearity (LIN = VSL/VCL x 100), straightness (STR = VSL/VAP x 100), and wobble (WOB = VAP/VCL x 100). A minimum of three fields and 500 sperm tracks were evaluated at a magnification of 100x for each sample (image acquisition rate 25 frames/s).

Propidium iodide (PI) and SYBR-14 were used as fluorochromes in the examination of membrane integrity [36]; 200 cells were examined using an epifluorescence microscope at 400× (wavelength: 450–490 nm).

All sperm variables were measured again for each pool after their eventual thawing. In addition, DNA integrity was also assessed in fresh sperm and after freezing-thawing by terminal deoxynucleotidyl transferase dUTP nick end labelling (TUNEL). For this, the kit “In Situ Cell Death Detection” (Roche, Basel, Switzerland) was used following manufacturer’s instructions with minor changes in order to adapt the technique to the analyses of rooster sperm. Briefly, each sperm sample was diluted to 12 x 10^6^ spermatozoa/mL in 4% paraformaldehyde. Subsequently 10 μL of this dilution were placed on a glass slide and left to dry. Then, the spermatozoa were permeabilized with 0.1% of Triton X-100 in PBS. After a wash in PBS, fragmented DNA was nick end-labelled with tetramethylrhodamine-conjugated dUTP by adding 10 μL of the working solution provided by the kit–containing the substrates and the enzyme terminal transferase–on the sample. The reaction was conducted incubating the slides in a humid box for 1 h at 37°C. After a wash with PBS the nucleus were counterstained with Hoechst at 0.1mg/mL in PBS for 5 min in the dark. Following an additional wash with PBS the slides were mounted using Fluoromount (Sigma-Aldrich, MO, USA) and observed under fluorescent microscopy (Eclipse E200, Nikon, Japan). Percentages of positive TUNEL spermatozoa (TUNEL+) per sample were recorded by counting a minimum of 200 spermatozoa per microscopy preparation, using an epifluorescence microscope at 400× (wavelength: 510–560 nm).

### Cryoprotectant removal and artificial insemination

White Prat and Black-Red Andaluza breeds were chosen for the insemination trial as examples of ‘good freezers’ and ‘bad freezers’, respectively on the basis of the post-thaw in vitro sperm assessments. Glycerol was removed prior to AI. Straws of semen frozen were thawed, and the thawed semen was progressively diluted with four volume parts of Lake Centri medium at 5°C by successively adding 0.07, 0.18, 0.33, 0.6, 1.24, and 1.58 volumes of medium to one volume of semen (2 min intervals). These samples were then centrifuged at 600xg for 10 min, the supernatant solution discarded, and the pellet resuspended (to the original volume of the thawed semen) in Lake and Ravie medium (method adapted from Mocé et al. [37]). All inseminations (see above) were performed between 12:00 h and 14:00 h. AI procedures involved 300 million sperm /female at each insemination. Eggs were collected from day two after the first AI until 3 days after the second AI. Fertility (% fertile/incubated eggs) was determined by candling the eggs (n = 144) on day 7 of incubation.

### Statistical analyses

Clustering by the amino acid content in seminal plasma of each breed was performed using the iterative k-technique to classify the amino acids into three clusters. Statistica software (TIBCO Software Inc. Palo Alto, CA, USA) specifically uses Lloyd's method to implement the k-Means algorithm [38]. The right number of clusters was determined by a v-fold cross-validation algorithm included in the Statistica package. Briefly, this method divides the overall sample into a number of v folds (*v value*: The default value is 10, the minimum is 2, and the maximum is 999). The same type of analysis is then successively applied to the observations belonging to the v-1 folds (training sample), and the results of the analyses are applied to sample v (testing sample) to compute an index of "predictive validity". Variables with a skewed distribution were arcsine-transformed (sperm variables), log-transformed (proteins) or submitted to box-cox transformation (amino acids) before statistical analysis. The influence of breed on amino acid and total protein were analysed by one way ANOVA, following the statistical model xij = m + Ai + eij, where xij = the measured variable (amino acid or total protein), m = the overall mean of x, Ai = the effect of breed (i = 1–12), and eij = the residual (j = 1–4). A Tukey post hoc analysis was performed to compare the differences between means of amino acids. Correlations between amino acids and sperm TUNEL+ and between amino acids and sperm viability were determined by the Spearman test; data of all breeds were included in the correlation analysis. The influence of breed and seminal plasma on frozen-thawed sperm variables were analysed by ANOVA, following the statistical model xijk = m + Ai + Bj + ABij + eijk, where xijk = the measured sperm variable, m = the overall mean of variable x, Ai = the effect of breed (i = 1–12), Bj = the effect of seminal plasma (j = 1–2), ABij = the interaction between A and B, and eijk = the residual (k = 1–14). A post hoc Newman-Keuls analysis was performed to compare the differences in mean sperm variable values between breeds and treatments (with and w/o seminal plasma). Comparisons between fresh and frozen-thawed sperm variables were made using a paired *t-*test. Identification of good and bad freezer was made by clustering (k-means cluster analysis; see above) the differences between percentage of sperm viability before and after freezing of each breed. The association among fertility rate and semen from good and bad freezers was assessed using the Chi-squared test. Data were expressed as means ± S.E. All statistical calculations were made using TIBCO Statistica software v.13.3 (TIBCO Software Inc.).

## Results

### Seminal plasma amino acids, total protein content and sperm concentration in different Spanish local chicken breeds

**Fig 1:**
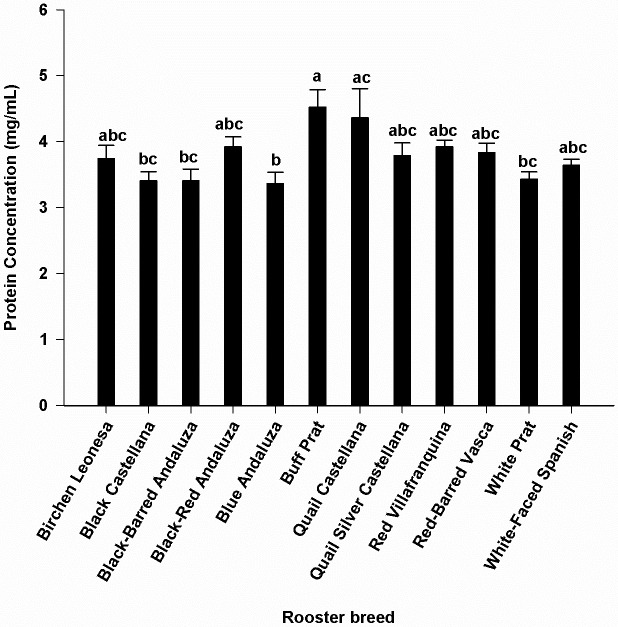
Comparison of seminal plasma protein concentrations (mean ± SE) between different breeds of chicken. Different letters (a,b,c) indicate significant differences (p<0.05). N = 48 (4 samples/breed).

Seminal plasma content in amino acid in each breed is shown in Table 1. Glutamic acid was by far the most abundant free amino acid in seminal plasma, accounting on average for more than 80% of the total free amino acid molar content, its concentration being the highest (P<0.001). Next to glutamine, the most abundant amino acids present in all breeds were alanine, serine, valine, and glycine. Proline was relatively abundant in some breeds, but was below detection limits in other breeds. Tryptophan was absent, or was present only in trace quantities. The highest (P<0.05) concentration of alanine, proline, cysteine and arginine were observed in Black-red Andaluza, Birchen Leonesa, White-Faced Spanish and Quail Silver Castellana, respectively. Buff Prat breed showed the lowest concentrations in these amino acids. Grouping the amino acid in three clusters, within each breed, revealed that some breeds showed the same pattern (Table 2). There was an effect of breed (P<0.05) on the seminal plasma concentrations of total proteins. Significant differences (P<0.05) were found between Buff Prat (the highest value) and Blue Andaluza, Black Castellana, Black-Barred Andaluza, White Prat breeds (the lowest values) (Fig 1). Statistical analysis on sperm concentration revealed significant differences (P< 0.05) between Red-Barred Vasca (3556.3 x 10^6^ spermatozoa/mL) and Quail Silver Castellana (1515.8 x 10^6^ spermatozoa/mL). The overall mean sperm concentration was 2204.9 (± 107.03) x 10^6^ spermatozoa/mL. No correlation was found between plasma total protein concentration and sperm concentration.

**Table 1:** Comparison of seminal plasma free amino acid concentrations (mean, range) between different breeds of chicken.

Free amino acids (mM)	Black-Red Andaluza	White-Faced Spanish	Quail Castellana	Quail Silver Castellana	Black-Barred Andaluza	Buff Prat	White Prat	Birchen Leonesa	Red-Barred Vasca	Black Castellana	Red Villafranquina	Blue Andaluza
Asp	0.55 (0.40–0.82)	0.63 (0.46–0.94)	0.56 (0.28–0.80)	0.56 (0.48–0.62)	0.55 (0.18–0.94)	0.34 (0.26–0.50)	0.34 (0.28–0.42)	0.63 (0.40–0.90)	0.47 (0.34–0.56)	0.52 (0.39–0.70)	0.43 (0.27–0.56)	0.74 (0.30–1.36)
Thr	0.68 (0.58–0.92)	1.03 (0.80–1.50)	0.71 (0.42–0.98)	0.77 (0.38–1.42)	0.96 (0.26–1.68)	0.45 (0.23–0.56)	0.61 (0.42–0.96)	0.80 (0.62–1.02)	0.61 (0.22–1.02)	0.44 (0.20–0.58)	0.51 (0.17–1.08)	0.59 (0.48–0.70)
Ser	1.41 (1.28–1.64)	1.43 (1.36–1.54)	1.48 (1.04–1.84)	1.55 (1.42–1.68)	1.14 (0.58–1.40)	0.86 (0.40–1.14)	1.36 (1.18–1.60)	1.40 (1.22–1.64)	1.14 (0.42–1.48)	0.82 (0.35–1.18)	0.80 (0.32–1.28)	1.23 (0.66–1.62)
Glu	49,70 (39.14–66.54)	51.17 (41.52–58.90)	45.13 (38.66–49.54)	36.48 (21.24–44.82)	61.12 (47.6–94.64)	40.03 (29.15–55.58)	32.60 (21.38–39.72)	43.84 (23.92–66.54)	37.81 (27.55–44.14)	47.36 (29.55–66.10)	39.02 (27.39–52.08)	54.46 (28.22–85.80)
Gly	1.01 (0.88–1.18)	1.05 (0.96–1.18)	0.95 (0.72–1.22)	0.97 (0.78–1.28)	0.88 (0.36–1.18)	0.58 (0.28–0.80)	0.99 (0.76–1.34)	0.99 (0.82–1.22)	0.77 (0.31–1.00)	0.57 (0.25–0.74)	0.66 (0.30–1.02)	0.91 (0.84–1.00)
Ala	1.73^a^ (1.54–1.90)	1.59 (1.42–1.72)	1.37 (1.00–1.62)	1.40 (1.34–1.52)	1.23 (0.72–1.52)	0.99^b^ (0.42–1.38)	1.34 (1.20–1.52)	1.53 (1.24–1.72)	1.17 (0.43–1.54)	0.90^b^ (0.39–1.24)	0.82^b^ (0.33–1.36)	1.32 (0.60–1.65)
Cys	0.13 (0.00–0.28)	0.32^a^ (0.28–0.40)	0.26^ab^ (0.20–0.32)	0.26^ab^ (0.22–0.32)	0.25^ab^ (0.18–0.30)	0.00^c^ (0.00–0.00)	0.27^ab^ (0.24–0.28)	0.26^ab^ (0.24–0.30)	0.06^bc^ (0.00–0.24)	0.17 (0.00–0.26)	0.10^bc^ (0.00–0.28)	0.27^ab^ (0.24–0.36)
Val	1.22 (0.90–1.44)	1.07 (0.98–1.12)	1.06 (0.68–1.42)	1.26 (1.00–1.44)	0.65 (0.28–0.96)	0.89 (0.40–1.20)	1.04 (0.94–1.20)	0.96 (0.66–1.20)	0.89 (0.45–1.16)	0.70 (0.45–0.90)	0.74 (0.31–1.18)	0.84 (0.48–1.12)
Met	0.30 (0.22–0.38)	0.30 (0.16–0.38)	0.25 (0.08–0.34)	0.34 (0.30–0.38)	0.14 (0.04–0.30)	0.19 (0.02–0.36)	0.32 (0.30–0.36)	0.24 (0.12–0.32)	0.18 (0.12–0.26)	0.20 (0.08–0.28)	0.19 (0.10–0.28)	0.14 (0.00–0.28)
Ile	0.32 (0.20–0.48)	0.36 (0.26–0.46)	0.37 (0.18–0.58)	0.41 (0.32–0.56)	0.26 (0.12–0.40)	0.15 (0.04–0.32)	0.33 (0.22–0.38)	0.26 (0.18–0.36)	0.19 (0.08–0.30)	0.13 (0.04–0.20)	0.22 (0.04–0.46)	0.23 (0.06–0.34)
Leu	0.53 (0.44–0.68)	0.54 (0.46–0.64)	0.61 (0.42–0.88)	0.66 (0.46–0.90)	0.40 (0.18–0.50)	0.30 (0.14–0.42)	0.52 (0.38–0.64)	0.51 (0.44–0.56)	0.37 (0.12–0.52)	0.27 (0.12–0.38)	0.30 (0.09–0.56)	0.43 (0.14–0.62)
Tyr	0.22 (0.18–0.28)	0.25 (0.22–0.30)	0.27 (0.18–0.36)	0.33 (0.24–0.42)	0.21 (0.10–0.30)	0.15 (0.06–0.20)	0.27 (0.22–0.30)	0.28 (0.24–0.34)	0.18 (0.06–0.22)	0.12 (0.05–0.20)	0.13 (0.03–0.26)	0.26 (0.06–0.42)
Phe	0.34 (0.26–0.40)	0.34 (0.30–0.38)	0.34 (0.24–0.44)	0.40 (0.32–0.58)	0.32 (0.26–0.38)	0.19 (0.12–0.22)	0.32 (0.24–0.44)	0.35 (0.30–0.38)	0.27 (0.10–0.36)	0.24 (0.11–0.34)	0.20 (0.00–0.36)	0.25 (0.00–0.40)
His	0.33 (0.26–0.38)	0.41 (0.34–0.50)	0.27 (0.20–0.30)	0.35 (0.32–0.36)	0.26 (0.14–0.34)	0.22 (0.08–0.30)	0.34 (0.32–0.34)	0.38 (0.28–0.42)	0.31 (0.08–0.42)	0.19 (0.06–0.28)	0.21 (0.07–0.36)	0.32 (0.08–0.46)
Lys	0.17 (0.12–0.22)	0.32 (0.20–0.62)	0.23 (0.10–0.36)	0.30 (0.14–0.42)	0.23 (0.04–0.42)	0.14 (0.06–0.20)	0.30 (0.26–0.38)	0.25 (0.20–0.38)	0.14 (0.06–0.20)	0.17 (0.10–0.20)	0.21 (0.06–0.48)	0.32 (0.10–0.66)
Arg	0.37 (0.28–0.44)	0.59 (0.50–0.64)	0.63 (0.30–1.00)	0.73^a^ (0.44–0.90)	0.32^c^ (0.10–0.42)	0.29^c^ (0.26–0.32)	0.61 (0.40–0.76)	0.55 (0.44–0.62)	0.37 (0.20–0.46)	0.30^bc^ (0.19–0.36)	0.26^c^ (0.11–0.44)	0.70^ab^ (0.48–0.84)
Pro	0.91 (0.00–1.31)	0.32 (0.00–1.28)	0.72 (0.00–1.19)	1.21 (0.00–2.50)	0.00^b^ (0.00–0.00)	0.01^b^ (0.00–0.04)	1.13 (0.91–1.60)	1.36^a^ (0.99–2.20)	0.62 (0.00–1.33)	0.00^b^ (0.00–0.00)	0.00^b^ (0.00–0.00)	0.67 (0.00–1.47)
Total	59.88 (47.06–77.97)	61.68 (50.76–70.62)	55.19 (45.66–60.83)	47.94 (36.18–53.02)	68.86 (56.60–103.06)	45.76 (32.17–62.80)	42.66 (33.66–48.84)	54.54 (32.63–78.98)	45.52 (30.54–54.01)	53.12 (32.33–73.06)	44.78 (29.97–60.32)	63.643 (37.81–92.42)

Aspartic acid (Asp), Threonine (Thr), Serine (Ser), Glutamic acid (Glu), Glycine (Gly), Alanine (Ala), Cysteine (Cys), Valine (Val), Methionine (Met), Isoleucine (Ile), Leucine (Leu), Tyrosine (Tyr), Phenylalanine (Phe), Histidine (His), Lysine (Lys), Arginine (Arg), Proline (Pro). N = 48 (4 samples/breed).

^a,b,c^ Different letters within rows indicate significant differences between breeds.

**Table 2:** Clusters of seminal plasma free amino acid concentrations in different breeds of chicken. Amino acid concentrations in cluster 1 > cluster 2 > cluster 3. Similar number of asterisks for each breed indicates the same amino acids in the three clusters.

Breed	Cluster 1	Cluster 2	Cluster 3
Black-Red Andaluza*	Glu	Ser, Gly, Ala, Val, Pro	Asp, Thr, Cys, Met, Ile, Leu, Tyr, Phe, His, Lis, Arg
Quail Silver Castellana*	Glu	Ser, Gly, Ala, Val, Pro	Asp, Thr, Cys, Met, Ile, Leu, Tyr, Phe, His, Lys, Arg
White Prat*	Glu	Ser, Gly, Ala, Val, Pro	Asp, Thr, Cys, Met, Ile, Leu, Tyr, Phe, His, Lys, Arg
Birchen Leonesa*	Glu	Ser, Gly, Ala, Val, Pro	Asp, Thr,Cys, Met, Ile, Leu, Tyr, Phe, His, Lys, Arg
Blue Andaluza*	Glu	Ser, Gly, Ala, Val, Pro	Asp, Thr, Cys, Met, Ile, Leu, Tyr, Phe, His, Lys, Arg
Quail Castellana**	Glu	Ser, Gly, Ala, Val	Asp, Thr, Cys, Met, Ile, Leu, Tyr, Phe, His, Lys, Arg, Pro
Buff Prat**	Glu	Ser, Gly, Ala, Val	Asp, Thr, Cys, Met, Ile, leu, Tyr, Phe, His, Lys, Arg, Pro
White-Faced Spanish***	Glu	Thr, Ser, Gly, Ala, Val	Asp, Cys, Met, Ile, Leu, Tyr, Phe, His, Lys, Arg, Pro
Red Villafranquina***	Glu	Thr, Ser, Gly, Ala, Val	Asp, Cys, Met, Ile, Leu, Tyr, Phe, His, Lys, Arg, Pro
Black Castellana	Glu	Asp, Thr, Ser, Gly, Ala, Val	Cys, Met, Ile, Leu, Tyr, Phe, His, Lys, Arg, Pro
Black-Barred Andaluza	Glu	Thr, Ser, Gly, Ala	Asp, Cys, Val, Met, Ile, Leu,Tyr, Phe, His, Lys, Arg, Pro
Red-Barred Vasca	Glu	Thr, Ser, Gly, Ala, Val, Pro	Asp, Cys, Met, Ile, leu, Tyr, Phe, His, Lys, Arg

Aspartic acid (Asp), Threonine (Thr), Serine (Ser), Glutamic acid (Glu), Glycine (Gly), Alanine (Ala), Cysteine (Cys), Valine (Val), Methionine (Met), Isoleucine (Ile), Leucine (Leu), Tyrosine (Tyr), Phenylalanine (Phe), Histidine (His), Lysine (Lys), Arginine (Arg), Proline (Pro). N = 48 (4samples/breed).

### Sperm viability

**Fig 2:**
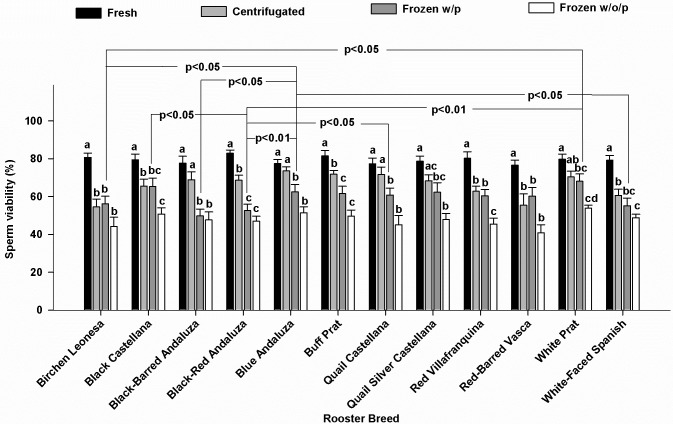
Viable sperm in fresh samples, after centrifugation and frozen-thawed samples without (w/o/p) or with (w/p) seminal plasma. Different letters (a,b) within each breed, indicates significant differences (p<0,05). Lines over bars (Frozen w/p) indicate significant differences between breeds (p<0,05; p<0,01). N = 168 (14 samples/breed).

There were no significant differences in fresh sperm viability between breeds. The removal of the seminal plasma by centrifugation reduced the sperm viability in seven of the 12 breeds (Black-Red Andaluza, White-Faced Spanish, Buff Prat, Birchen Leonesa, Red-Barred Vasca, Black Castellana, and Red Villafranquina; Fig 2). Despite the negative effect of centrifugation in seven breeds, the post-thaw sperm viability was not significantly affected by the removal of seminal plasma in all breeds, with the only exception of Red Villafranquina breed (P< 0.05) (Fig 2). There were no significant differences between breeds in the percentage of viable sperm after freezing-thawing in samples without seminal plasma. In contrast, a significant effect of the breed was found (P<0.05) on the percentage of viable sperm after freezing-thawing in samples with seminal plasma. Cluster analysis in these last samples, carried out with the values obtained from the differences between the percentages of sperm viability before freezing and after thawing of each breed, revealed that White Prat, Black Castellana, Blue Andaluza, Quail Castellana, and Red-Barred Vasca returned the best freezing-thawing response (good freezers: mean sperm viability after freezing-thawing in samples with seminal plasma: 63.4±1.5%; difference between fresh and post-thawed sperm viability with plasma: 14.7±0.9%), whereas the worst response was found in Black-Red Andaluza, Black-Barred Andaluza, White-Faced Spanish and Buff Prat (bad freezers: mean sperm viability after freezing-thawing in samples with seminal plasma: 54.8±2.5%; difference between fresh and post-thawed sperm viability with plasma: 25.6±2.3%).

**Fig 3:**
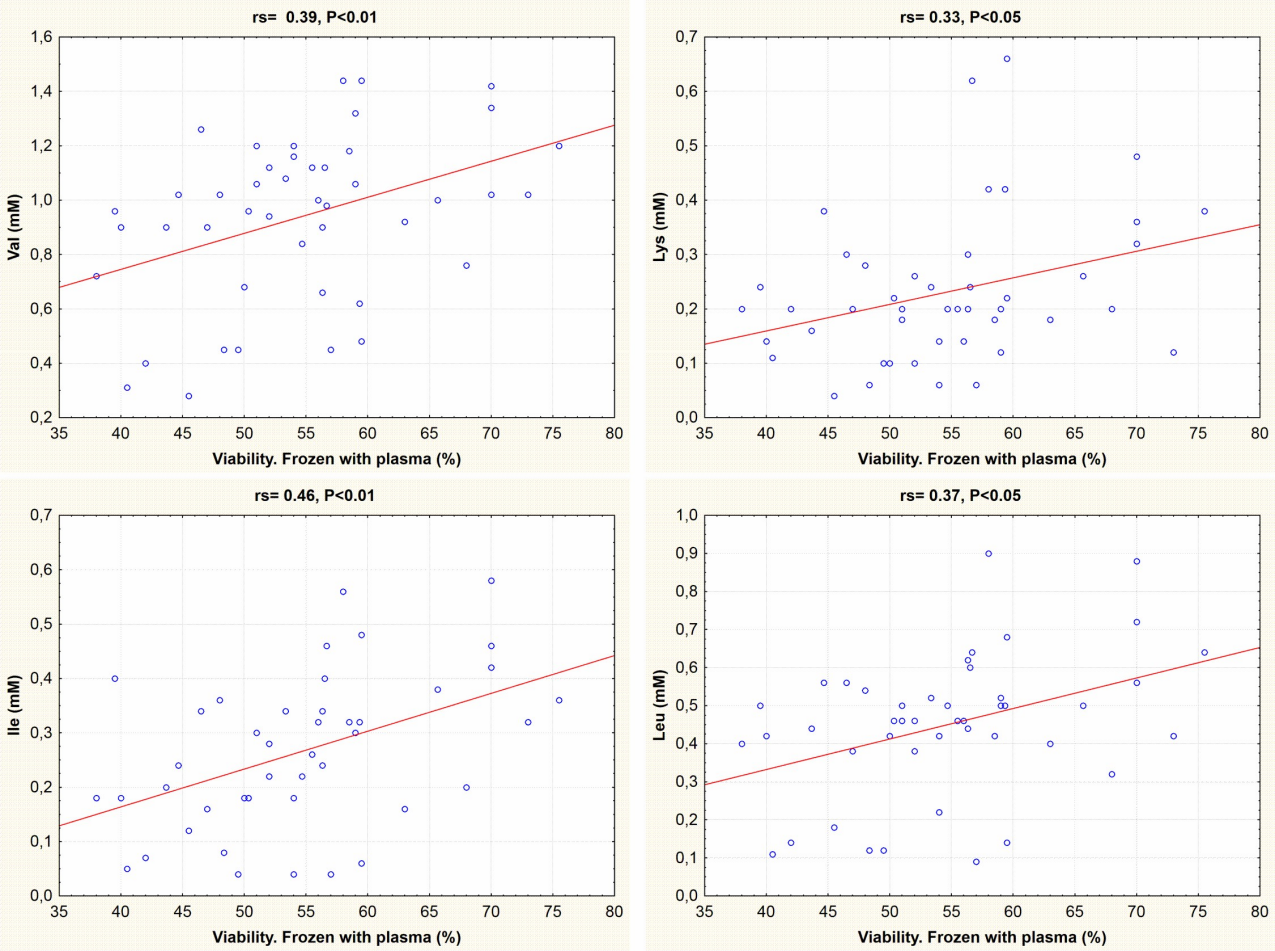
Correlation between sperm viability and amino acids concentrations. Sperm viability corresponds to the percentage of sperm with intact membrane in frozen-thawed samples with seminal plasma. The amino acids concetration includes all chicken breeds. rs, Spearman rank correlation coefficient.

The differences in sperm freezing-thawing response were not correlated with protein concentrations. In frozen-thawed semen samples with seminal plasma, there was a positive correlation between sperm viability and the concentrations of valine, isoleucine, lysine and leucine (Fig 3).

### Sperm motility

**Fig 4:**
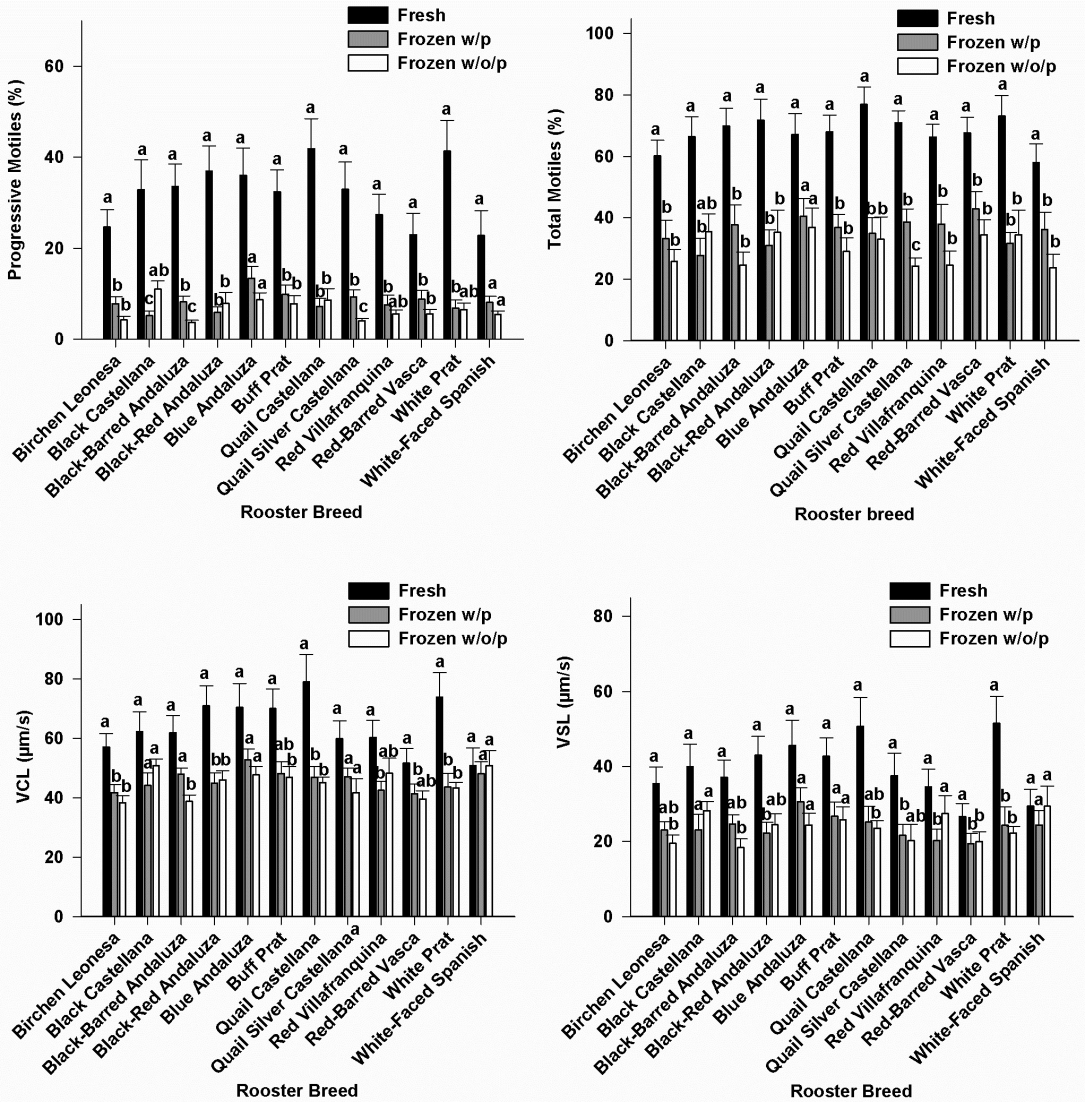
Motility sperm variables in fresh and frozen-thawed samples without (w/o/p) or with (w/p) seminal plasma. Different letters (a,b) within each breed indicates significant differences (p<0,05). N = 168 (14 samples/breed).

The removal of seminal plasma did not significantly affected sperm motion parameters measured in frozen-thawed samples in the majority of breeds. In particular, the removal of seminal plasma significantly decreased progressive motility (P<0.05) and VCL (P<0.05) in Black-Barred Andaluza, and total (P<0.05) and progressive motility (P<0.05) in Quail Silver Castellana; in contrast, the removal of seminal plasma significantly increased VSL (P<0.05) in Red Villafranquina (Fig 4). Significant correlations between amino acid concentrations and motility sperm variables were not found.

### Sperm DNA fragmentation

**Fig 5:**
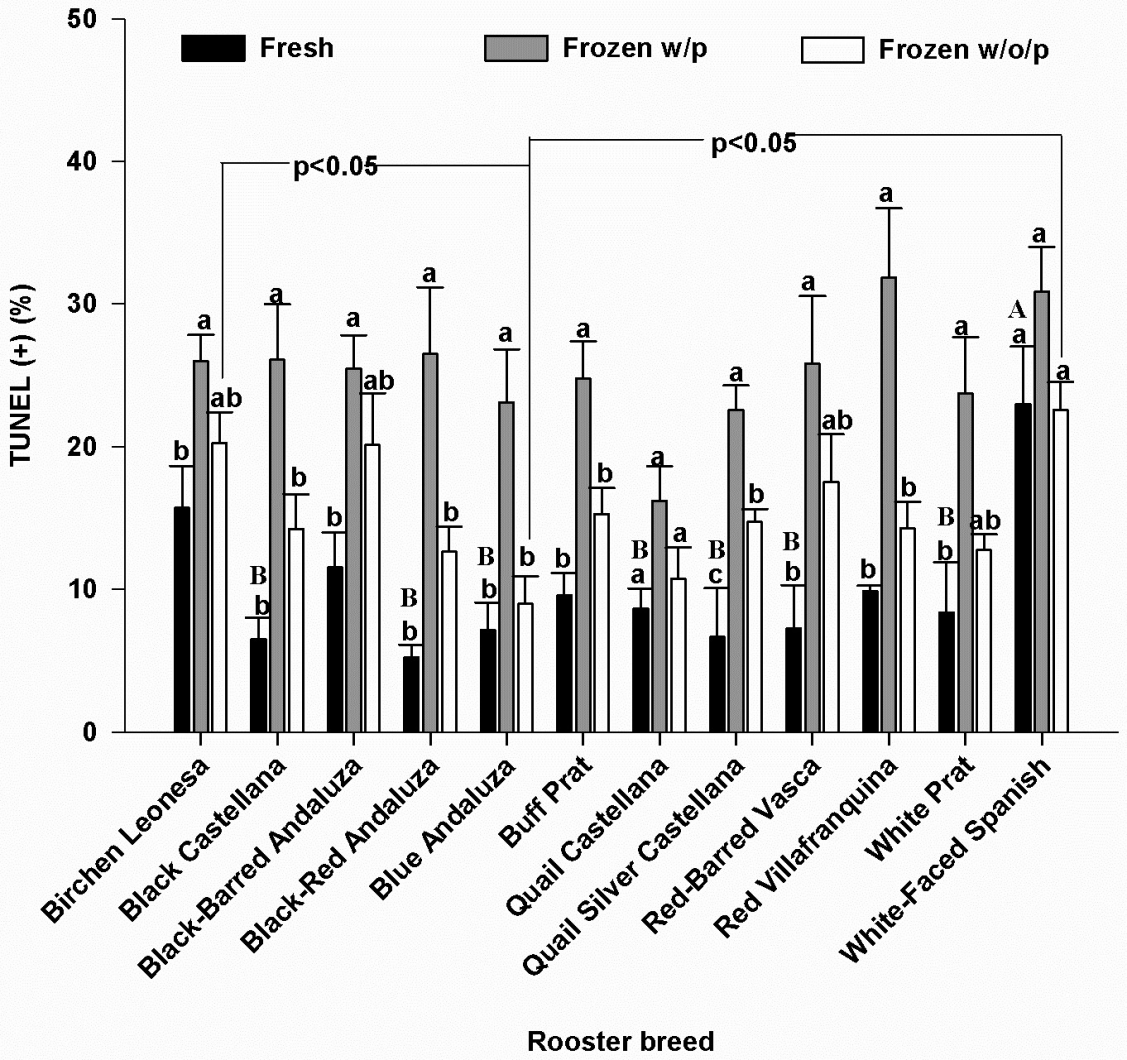
TUNEL + in fresh and frozen-thawed samples without (w/o/p) or with (w/p) seminal plasma. Different letters (a,b) within each breed indicate significant differences (P<0,05). Different letters (A,B) between breeds indicate significant differences (P<0,05) in fresh samples. Lines over bars (Frozen w/o/p) indicate significant differences between breeds. N = 168 (14 samples/breed).

The TUNEL assay revealed that fresh sperm from White-Faced Spanish had the highest degree of DNA fragmentation being statistically higher (P<0.05) than most of the breeds (Fig 5). We found that the presence of seminal plasma increased DNA fragmentation during freezing/thawing in most of the breeds (Fig 5). Only in Quail Castellana breed (with low DNA fragmentation in fresh sperm) and White-Faced Spanish (with high DNA fragmentation in fresh sperm) there were no differences in the percentage of TUNEL + sperm between fresh and frozen samples with seminal plasma (Fig 5). DNA fragmentation in Blue Andaluza was less (P<0.05) than in White-Faced Spanish and Birchen Leonesa for samples cryopreserved without seminal plasma.

**Fig 6:**
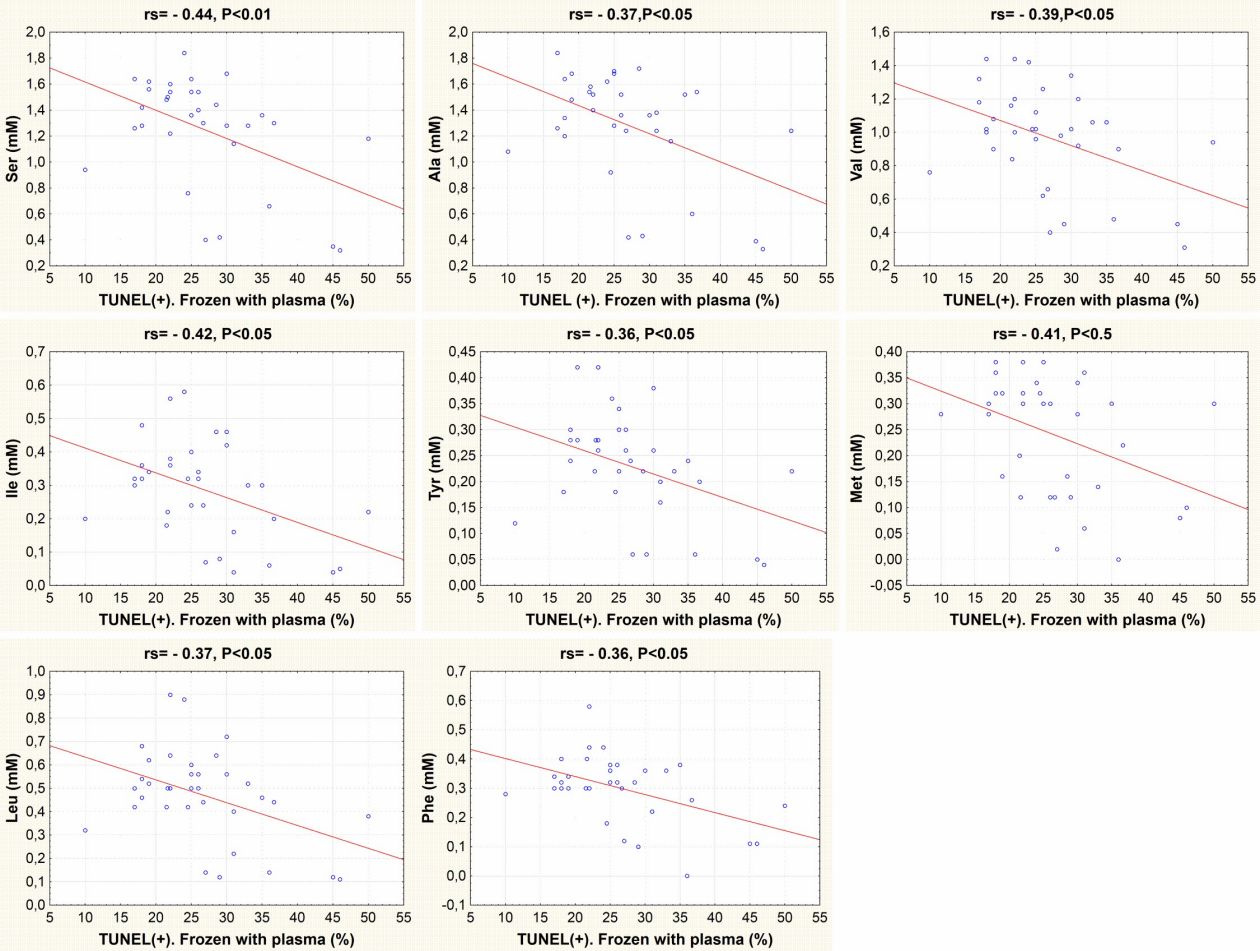
Correlation between sperm TUNEL + and amino acids concentrations. TUNEL + corresponds to the percentage of sperm with DNA fragmentation in frozen-thawed samples with seminal plasma. The amino acids concetration includes all chicken breeds. rs, Spearman rank correlation coefficient.

In fresh samples, cysteine was the only amino acid with a positive correlation with % TUNEL+ sperm (i.e. DNA damaged sperm; rs = 0.43, P<0.05). In frozen-thawed semen samples with plasma, there was a negative correlation between the values of post-thaw TUNEL+ and certain amino acids such as alanine, valine, isoleucine, methionine, leucine, tyrosine, phenylalanine, serine (i.e. a positive correlation of the concentration of these amino acids with integrity of DNA) (Fig 6).

### Fertility

The evaluation of fertilization capacity of frozen-thawed semen from the breeds White Prat and Black-Red Andaluza, as examples of ‘good freezers’ and ‘bad freezers’, respectively, showed that the good freezer had higher fertility (20/68, 29.4%) compared to bad freezer breed (14/76, 18.4%), even if the difference was not significant (P = 0.08).

## Discussion

In the present study, we showed that seminal plasma and breed differences in seminal amino acids contents affect the results of chicken sperm cryopreservation. There was a relationship between concentrations of some amino acids (i.e. valine and leucine that are common for all criteria) and sperm viability and DNA integrity after freezing-thawing irrespective of the breed. Although the deleterious effect of freezing-thawing on the DNA integrity of chicken sperm has been previously reported [39, 40, 41], herein we show for the first time that there is differential susceptibility of the DNA to the cryoinjury depending on the chicken breed. Despite a frequent harmful effect of centrifugation, the removal of seminal plasma before freezing allowed to decrease the DNA fragmentation damages induced by cryopreservation and allowed to reduce the breed variability effect on post-thaw sperm viability.

Differences between breeds were found with respect to seminal free amino acid concentrations, and some of these differences were relevant regarding sperm cryoprotection. Glutamic acid was the main amino acid in seminal plasma, accounting for 76–89% (on molar basis) of the total amino acid content. This is in agreement with earlier observations in cock semen [42]. Glutamate is thought to serve as the main anion in place of Cl- [43]. In addition, glutamate may act as a motility agonist when sperm is co-incubated with Ca2+ under aerobic conditions [44]; it has been suggested that rooster sperm express glutamate channels that mediate the flux of Ca2+ and K+ at the mitochondria membrane levels, and hence contributing to sperm kinetics [44,45]. We didn’t find any relationship between glutamate and motility variables between breeds. The other amino acids were present in much lower concentrations. Excluding glutamic acid, Ahluwalia and Graham [46] reported that arginine, asparagine, threonine, and glycine are the most prevalent in chicken seminal plasma of mixed breed. Aspartic acid is the second most abundant amino acid in Delaware and New Hampshire rooster [47], and in Brown Leghorn [48]. Our data showed that apart from glutamine, the most abundant amino acids present in all breeds studied here were alanine, serine, valine, and glycine, but that there were different clusters of specific seminal plasma amino acids levels depending on the breed. This highlights the role of genetic components (breed differences) on the free amino acid concentrations of rooster seminal plasma.

The use of glutamine as a component of the freezing medium resulted in a higher post-thaw motility in human [23] and rooster [49] sperm. Glutamine, glycine and cysteine as additives in conventional freezing medium enhanced post-thaw motility and improved membrane and acrosome integrity of buffalo bull semen [50]. Addition of glutamine and proline improved sperm motility variables, membrane and acrosome integrity in frozen-thawed goat sperm [51]. Although the cryoprotectant mechanism of amino acids is not well known, some hypotheses have been provided in this way. Amino acids might interact with phospholipids bilayers during freezing [52] allowing stabilizing the cell membrane. In addition, they might protect sperm during freezing by colligative action through their unspecific ability to reduce the concentration of toxic solutes below the limit of toxicity [53]. Some amino acids, such as proline, might act as a solute protecting the cell against the denaturing effects of hyperosmolality induced by dehydration during slow freezing [26].

We found a positive association between the seminal plasma concentration of the hydrophobic amino acids valine, isoleucine and leucine and the charged lysine with membrane integrity as measured by the viability assay. It remains unknown whether these associations point to a casual effect, i.e. a cryoprotective effect of these amino acids. This could be tested in future experiments by including these amino acids in varying concentrations in the freezing medium.

There were no significant differences between breeds in the percentage of sperm viability after freezing-thawing of samples without seminal plasma, whereas there was an influence of breed on the percentage of viable sperm after freezing-thawing of samples with seminal plasma. This suggests a role of seminal plasma to prevent or to favor cryoinjury during freezing-thawing process. The influence of factors that may vary between breeds other than seminal plasma amino acids should not be ruled out. For instance, Blesbois and de Reviers [8], reported that low molecular weight seminal plasma fractions can reduce the fertilizing ability of sperm during storage at 4°C, whereas high molecular weight fractions appeared to enhance fertilizing ability. Seminal plasma is involved in degradation of sperm phospholipids, possibly by phospholipase activity, accelerating the sperm damage of turkey sperm during in vitro storage [10]. Genetic variations in seminal plasma proteins expression [14, 54] or other plasma components, may thus affect the freezability of the semen of a breed.

The decreasing of sperm viability in samples frozen without plasma seems mainly due to damage during centrifugation. Interestingly, we found that, whereas the presence of seminal plasma did not seem to affect the sperm survival (% viable), as the relative decrease of viability, i.e. post-thaw compared with pre-freeze, was not different between semen frozen with and without plasma, the DNA was less damaged in the latter. This could reflect different mechanisms of damage; plasma membrane integrity may predominantly be damaged by temperature- and dehydration-dependent membrane phase transitions [55], along with mechanical forces associated with ice formation and shrinking of the cells during freezing, while DNA might be more sensitive to the generation of reactive oxygen species (ROS) [56]. Possibly, this latter mechanism is stimulated by the presence of seminal plasma. The overproduction of ROS which exceeds the seminal plasma antioxidant capacity disturbs the balance between seminal ROS and antioxidant capacity, and results in oxidative stress. Oxidative damage may originate from several potential resources from seminal plasma, such as leukocytes [57] and presence of immature sperm. In addition, our findings suggest that others non-identified components of the seminal plasma of chickens might stimulate excessive generation of ROS by sperm and/or strongly decrease the levels of antioxidant defenses [58] during freezing-thawing process.

Negative correlations were seen between post-thaw %TUNEL+ sperm frozen with seminal plasma and the concentrations in seminal plasma of specific amino acids: alanine, valine, isoleucine, methionine, leucine, tyrosine, phenylalanine, serine, most of which are of hydrophobic nature. Thus, it could be expected that incorporating some of these amino acids in freezing media could decrease DNA damage during freeze-thawing. It was indeed reported that the incorporation of methionine to bovine [59] and fish [60] freezing media reduced the loss of DNA integrity during freezing and thawing. Accordingly, supplementing chicken extenders with the non-coded amino acid taurine, showed also a positive effect in reducing sperm apoptosis and DNA damage [61]. Although cysteine concentrations weren’t correlated with DNA integrity in frozen-thawed sperm with seminal plasma, we found that cysteine was the only amino acid positively correlated with DNA damage in fresh samples, and White-Faced Spanish, the breed with the highest value of cysteine, showed the highest values of damage in DNA of samples in fresh plasma. However, supplementation of extenders with L-cysteine for the cryopreservation of carp sperm reported a decrease in DNA damage in post-thawed sperm [62] and incorporating cysteine to buffalo extenders did not significantly affect the DNA damage [63]. Thus, the protective effect of the amino acids on DNA during cryopreservation might vary among species, possibly related to the different packaging and ultrastructure of the chromatin. For example, carp [64] and other fish species [65] maintain a nucleosome organization of the sperm chromatin whereas in mammals and birds [65, 66] there is a substitution of the histones for protamines during spermatogenesis in order to increase the chromatin compaction and the protection of the DNA. In addition, the chromatin of rooster sperm contains no cysteine residues [65] and lacks the potential stabilizing effect of S-S bonds of mammalian sperm chromatin.

The seminal plasma protein concentrations differed between breeds, in agreement with previous studies in other chicken breeds [47]. While significant differences were found between sperm concentration of two of the twelve breeds studied, these sperm concentrations did not seem related to the respective total plasma protein concentration, and over all breeds there was no correlation between both variables. However, these differences did not seem related with the observed differences in sperm cryodamage.

In conclusion, the results suggest that the decreasing of sperm viability in samples frozen without seminal plasma is largely due to damage during centrifugation. Our findings indicate that removal of seminal plasma did not seem to affect sperm survival during freezing and thawing, but did clearly reduce DNA damage of sperm. Specific amino acids were associated with post-thaw percentage of viable sperm and DNA integrity.

## Supporting information

S1 Dataset Amino acids profile in seminal plasma of 12 Spanish rooster breeds. (PDF)

S2 Dataset Protein concentrations in seminal plasma of 12 Spanish rooster breeds. (PDF)

S3 Dataset Viability and concentration of fresh sperm. Sperm viability of frozen sperm with plasma and without plasma of 12 Spanish rooster breeds.
(PDF)

S4 Dataset Motility variables of sperm of 12 Spanish rooster breeds (fresh, frozen with plasma and frozen without plasma). (PDF)

S5 Dataset Tunel + of sperm of 12 Spanish rooster breeds (fresh, frozen without plasma and frozen with plasma). (PDF)
