# Supplementary material for: Seminal plasma amino acid profile in different breeds of chicken: Role of seminal plasma on sperm cryoresistance
Source: PLoS One. 2019 Jan 4;14(1):e0209910. doi: 10.1371/journal.pone.0209910 (PMC6319765; doi:10.1371/journal.pone.0209910)
Supplement: S1 Dataset — (PDF) [file pone.0209910.s001.pdf]

S1 Dataset. Amino acids profile in seminal plasma of 12 Spanish rooster breeds.

Rooster Breed	Month	Asp	Thr	Ser	Glu
Birchen Leonesa	August	0,9	0,74	1,22	66,54
Birchen Leonesa	September	0,48	0,62	1,44	32,02
Birchen Leonesa	October	0,74	1,02	1,64	52,88
Birchen Leonesa	November	0,4	0,8	1,3	23,92
Black Castellana	August	0,7	0,54	0,94	66,1
Black Castellana	September	0,48	0,58	1,18	46,42
Black Castellana	October	0,39	0,2	0,35	29,55
Black-Barred Andaluza	August	0,94	0,78	1,2	94,64
Black-Barred Andaluza	September	0,18	0,26	0,58	54,48
Black-Barred Andaluza	October	0,42	1,1	1,36	47,74
Black-Barred Andaluza	November	0,66	1,68	1,4	47,6
Black-Red Andaluza	August	0,82	0,64	1,4	66,54
Black-Red Andaluza	September	0,48	0,58	1,64	49,6
Black-Red Andaluza	October	0,4	0,58	1,3	39,14
Black-Red Andaluza	November	0,48	0,92	1,28	43,52
Blue Andaluza	August	0,3	0,58	1,12	28,22
Blue Andaluza	September	0,8	0,6	1,5	71,66
Blue Andaluza	October	0,48	0,7	1,62	32,14
Blue Andaluza	November	1,36	0,48	0,66	85,8
Buff Prat	August	0,5	0,5	1,14	55,58
Buff Prat	September	0,3	0,52	1,14	42,92
Buff Prat	October	0,3	0,23	0,4	29,15
Buff Prat	November	0,26	0,56	0,76	32,48
Quail Castellana	August	0,28	0,42	1,04	38,66
Quail Castellana	October	0,6	0,72	1,56	49,54
Quail Castellana	November	0,8	0,98	1,84	47,18
Quail Silver Castellana	August	0,6	0,6	1,54	41,6
Quail Silver Castellana	September	0,48	0,38	1,42	44,82
Quail Silver Castellana	October	0,62	0,68	1,68	38,26
Quail Silver Castellana	November	0,54	1,42	1,54	21,24
Red Villafranquina	August	0,37	0,17	0,32	32,86
Red Villafranquina	September	0,56	0,6	1,26	52,08
Red Villafranquina	October	0,27	0,18	0,32	27,39
Red Villafranquina	November	0,5	1,08	1,28	43,76
Red-Barred Vasca	August	0,56	0,64	1,36	41,22
Red-Barred Vasca	September	0,44	0,56	1,48	38,32
Red-Barred Vasca	October	0,34	0,22	0,42	27,55
Red-Barred Vasca	November	0,52	1,02	1,28	44,14
White Prat	August	0,36	0,44	1,18	31,92
White Prat	September	0,3	0,42	1,22	39,72
White Prat	October	0,42	0,62	1,42	37,38
White Prat	November	0,28	0,96	1,6	21,38
White-Faced Spanish	August	0,6	0,82	1,38	55,4
White-Faced Spanish	September	0,5	0,8	1,36	41,52
White-Faced Spanish	October	0,46	1	1,54	48,84
White-Faced Spanish	November	0,94	1,5	1,44	58,9

Gly	Ala	Cys	Val	Met	Ile	Leu
1,22	1,72	0,24	1,2	0,32	0,36	0,56
0,84	1,46	0,24	0,96	0,2	0,18	0,46
1,08	1,68	0,3	1,02	0,3	0,24	0,56
0,82	1,24	0,24	0,66	0,12	0,24	0,44
0,72	1,08	0,24	0,76	0,28	0,2	0,32
0,74	1,24	0,26	0,9	0,24	0,16	0,38
0,25	0,39	0	0,45	0,08	0,04	0,12
1,12	1,38	0,26	0,72	0,08	0,18	0,4
0,36	0,72	0,18	0,28	0,04	0,12	0,18
0,84	1,28	0,24	0,96	0,3	0,4	0,5
1,18	1,52	0,3	0,62	0,12	0,32	0,5
1,18	1,9	0,28	1,2	0,28	0,3	0,5
0,96	1,84	0	1,32	0,3	0,3	0,5
0,88	1,54	0,22	0,9	0,22	0,2	0,44
1	1,64	0	1,44	0,38	0,48	0,68
0,84	1,4	0,24	1,12	0,28	0,28	0,46
1	1,58	0,24	0,84	0,12	0,22	0,5
0,96	1,68	0,24	0,9	0,16	0,34	0,62
0,84	0,6	0,36	0,48	0	0,06	0,14
0,8	1,38	0	1,2	0,36	0,04	0,22
0,68	1,24	0	0,92	0,06	0,16	0,4
0,28	0,42	0	0,4	0,02	0,07	0,14
0,54	0,92	0	1,02	0,32	0,32	0,42
0,72	1	0,2	0,68	0,08	0,18	0,42
0,9	1,48	0,26	1,08	0,32	0,34	0,52
1,22	1,62	0,32	1,42	0,34	0,58	0,88
1	1,36	0,32	1,26	0,3	0,34	0,56
0,78	1,34	0,24	1	0,32	0,32	0,46
0,82	1,36	0,26	1,34	0,34	0,42	0,72
1,28	1,52	0,22	1,44	0,38	0,56	0,9
0,33	0,33	0	0,45	0,1	0,04	0,09
0,98	1,26	0	1,18	0,28	0,32	0,42
0,3	0,33	0,11	0,31	0,1	0,05	0,11
1,02	1,36	0,28	1,02	0,28	0,46	0,56
0,94	1,54	0,24	0,9	0,26	0,18	0,42
0,84	1,54	0	1,16	0,2	0,18	0,42
0,31	0,43	0	0,45	0,12	0,08	0,12
1	1,16	0	1,06	0,14	0,3	0,52
0,9	1,24	0,24	0,94	0,3	0,22	0,38
0,76	1,4	0,28	1	0,32	0,38	0,5
0,96	1,2	0,26	1,02	0,36	0,36	0,54
1,34	1,52	0,28	1,2	0,3	0,36	0,64
1,06	1,42	0,3	1,12	0,34	0,26	0,46
0,96	1,52	0,28	1,06	0,3	0,3	0,46
1	1,7	0,3	1,12	0,38	0,4	0,6
1,18	1,72	0,4	0,98	0,16	0,46	0,64

Tyr	Phe	His	Lys	Arg	Pro	Nleu
0,26	0,38	0,42	0,2	0,5	2,204	0
0,26	0,34	0,4	0,22	0,62	1,186	0
0,34	0,38	0,4	0,38	0,62	1,048	0
0,24	0,3	0,28	0,2	0,44	0,994	0
0,12	0,28	0,24	0,2	0,34	0	0
0,2	0,34	0,28	0,2	0,36	0	0
0,05	0,11	0,06	0,1	0,19	0	0
0,2	0,32	0,26	0,2	0,38	0	0
0,1	0,26	0,14	0,04	0,1	0	0
0,22	0,32	0,3	0,24	0,38	0	0
0,3	0,38	0,34	0,42	0,42	0	0
0,22	0,4	0,38	0,18	0,44	1,308	0
0,18	0,3	0,34	0,12	0,28	1,134	0
0,2	0,26	0,26	0,16	0,36	0	0
0,28	0,4	0,32	0,22	0,4	1,18	0
0,28	0,28	0,36	0,1	0,48	1,472	0
0,28	0,4	0,38	0,2	0,66	0	0
0,42	0,3	0,46	0,3	0,82	1,194	0
0,06	0	0,08	0,66	0,84	0	0
0,2	0,22	0,28	0,06	0,32	0	0
0,16	0,22	0,3	0,18	0,26	0	0
0,06	0,12	0,08	0,2	0,3	0	0
0,18	0,18	0,22	0,12	0,28	0,042	0
0,18	0,24	0,2	0,1	0,3	0,964	0
0,28	0,34	0,3	0,24	0,6	0	0
0,36	0,44	0,3	0,36	1	1,194	0
0,26	0,32	0,36	0,3	0,7	1,116	0
0,24	0,32	0,32	0,14	0,44	0	0
0,38	0,36	0,36	0,32	0,9	1,216	0
0,42	0,58	0,34	0,42	0,88	2,502	0
0,03	0	0,07	0,06	0,11	0	0
0,18	0,34	0,34	0,18	0,34	0	0
0,04	0,11	0,08	0,11	0,16	0	0
0,26	0,36	0,36	0,48	0,44	0	0
0,2	0,32	0,4	0,14	0,38	1,164	0
0,22	0,3	0,42	0,14	0,46	0	0
0,06	0,1	0,08	0,06	0,2	0	0
0,22	0,36	0,32	0,2	0,44	1,328	0
0,22	0,24	0,32	0,26	0,56	0,912	0
0,26	0,3	0,34	0,26	0,4	0,98	0
0,3	0,3	0,34	0,28	0,7	1,048	0
0,28	0,44	0,34	0,38	0,76	1,598	0
0,22	0,3	0,42	0,2	0,56	1,276	0
0,24	0,38	0,38	0,2	0,5	0	0
0,3	0,36	0,34	0,24	0,64	0	0
0,22	0,32	0,5	0,62	0,64	0	0
