# Supplementary material for: Seminal plasma amino acid profile in different breeds of chicken: Role of seminal plasma on sperm cryoresistance
Source: PLoS One. 2019 Jan 4;14(1):e0209910. doi: 10.1371/journal.pone.0209910 (PMC6319765; doi:10.1371/journal.pone.0209910)
Supplement: S2 Dataset — (PDF) [file pone.0209910.s002.pdf]

S2 Dataset. Protein concentrations in seminal plasma of 12 Spanish rooster breeds.

Rooster Breed	Protein Concentration (mg/mL)				
	June	July	August	September	October
Birchen Leonesa	3,14785	4,10765	3,96345	3,3696	4,11455
Black Castellana	3,70735	3,4175	3,1631	3,03425	3,70765
Black-Barred Andaluza	3,4418	3,6946	3,2306	2,8297	3,8298
Black-Red Andaluza	4,0689	3,65145	4,44285	3,62285	3,81825
Blue Andaluza	3,1684	2,91465	3,96115	3,41355	3,36475
Buff Prat	4,0964	4,0631	4,4855	4,43755	5,52995
Quail Castellana	5,6238	4,1807	3,5832		4,0581
Quail Silver Castellana	4,4345	3,7033	3,7519	3,225	3,82175
Red Villafranquina	3,8095	3,9543	4,23005	3,6352	3,9814
Red-Barred Vasca	3,6125	3,7554	3,5991	3,7861	4,39755
White Prat	3,27995	3,16945	3,3359	3,6643	3,7142
White-Faced Spanish	3,44055	3,9271	3,6419	3,4332	3,75635
