# Supplementary material for: Seminal plasma amino acid profile in different breeds of chicken: Role of seminal plasma on sperm cryoresistance
Source: PLoS One. 2019 Jan 4;14(1):e0209910. doi: 10.1371/journal.pone.0209910 (PMC6319765; doi:10.1371/journal.pone.0209910)
Supplement: S3 Dataset — Sperm viability of frozen sperm with plasma and without plasma of 12 Spanish rooster breeds. (PDF) [file pone.0209910.s003.pdf]

S3 Dataset. Viability and concentration of fresh sperm. Sperm viability of frozen sperm with plasma and without plasma of 12 Spanish rooster breeds.

w/p= with plasma w/o/p= without plasma

Rooster Breed	Date	Viability (%)				Concentration (10^6 spz/ml
		Fresh	Centrifugated	Frozen w/p	Frozen w/o/p	
Black-Red Andaluza	6/20/2016	86				4065,5
Black-Red Andaluza	6/27/2016	69		63		3887,3
Black-Red Andaluza	7/4/2016	83		34		4917,6
Black-Red Andaluza	7/13/2016	91				1137,5
Black-Red Andaluza	7/18/2016	88	79	56	51	6538,9
Black-Red Andaluza	8/10/2016	90	79	51	47	2597,6
Black-Red Andaluza	9/7/2016	83	58	68	46	4269,7
Black-Red Andaluza	9/19/2016	75	63	42	40	3581,5
Black-Red Andaluza	9/28/2016	86	68	67	52	1724
Black-Red Andaluza	10/10/2016	74	79	38	47	3765,6
Black-Red Andaluza	10/17/2016	80	66	31	47	1748,2
Black-Red Andaluza	10/26/2016	82	72	62	30	1232,3
Black-Red Andaluza	11/10/2016	83	74	61	67	564,5
Black-Red Andaluza	11/16/2016	90	48	58	44	1475,1
Birchen Leonesa	6/20/2016	87				1466
Birchen Leonesa	6/27/2016	77		70		2069,2
Birchen Leonesa	7/4/2016	85				6822
Birchen Leonesa	7/13/2016	81		84		4783,2
Birchen Leonesa	7/18/2016	82	48	66	26	2185,6
Birchen Leonesa	9/7/2016	78	62	54	61	3188,8
Birchen Leonesa	9/19/2016	75	57	41	60	3308,3
Birchen Leonesa	9/21/2016	85	75	56	49	1353,3
Birchen Leonesa	10/3/2016	80	80	63	44	1169,3
Birchen Leonesa	10/17/2016	70	38	32	2	1022,8
Birchen Leonesa	10/26/2016	61	31	39	51	1387,1
Birchen Leonesa	11/10/2016	86	48	43	45	943,9

Birchen Leonesa	11/16/2016	94	47	70	60	1205
Birchen Leonesa	11/21/2016	89	60	56	45	1034,4
Red-Barred Vasca	6/20/2016	86		80		2968,1
Red-Barred Vasca	6/27/2016	75		88		3600,9
Red-Barred Vasca	7/4/2016	69				3726,3
Red-Barred Vasca	7/13/2016	78		82	58	6680,3
Red-Barred Vasca	7/18/2016	85	61	66	53	2129,6
Red-Barred Vasca	8/10/2016	90	76	40	31	7022,6
Red-Barred Vasca	9/7/2016	76	63	67	45	5428,2
Red-Barred Vasca	9/19/2016	69	54	44	45	3602,7
Red-Barred Vasca	9/21/2016	69	0	51	63	2418,5
Red-Barred Vasca	10/3/2016	86	74	55	47	4389,1
Red-Barred Vasca	10/17/2016	74	62	57	32	516,7
Red-Barred Vasca	10/26/2016	53	54	33	37	2148,9
Red-Barred Vasca	11/7/2016	85	55	59	50	1599,5
Quail castellana	6/20/2016	88				1373,1
Quail castellana	6/27/2016	72		76		2051,2
Quail castellana	7/4/2016	64		68		2176,6
Quail castellana	7/13/2016	78		82		2061,9
Quail castellana	7/18/2016	49	73	45	71	2582
Quail castellana	8/10/2016	88	81	50	19	1314,2
Quail castellana	9/7/2016	89	68	70	40	3350,1
Quail castellana	9/19/2016	88	94	50	50	2807,5
Quail castellana	9/21/2016	74	69	50	43	1958,9
Quail castellana	10/3/2016	81	87	58	42	2923,2
Quail castellana	10/17/2016	71	44	38	17	1151,5
Quail castellana	10/26/2016	88	67	64	55	1159,4
Quail castellana	11/10/2016	70	54	75	43	842,3
Quail castellana	11/16/2016	82	79	65	71	1538,8
White Prat	6/20/2016	82		78		2164,6
White Prat	6/27/2016	78		79		2812,6
White Prat	7/4/2016			84		1280,9
White Prat	7/13/2016	74		81		3081,3

White Prat	7/18/2016	88	57	87	47	3538,3
White Prat	8/10/2016	76	76	52	54	2861,9
White Prat	9/7/2016	69	59	63	57	2364,7
White Prat	9/19/2016	88	52	72	53	726,2
White Prat	9/21/2016	93	67	62	56	1228,1
White Prat	10/3/2016	89	79	51	54	3303,9
White Prat	10/17/2016	54	80	41	46	1438,7
White Prat	10/26/2016	79	85	52	48	692
White Prat	11/10/2016	78	68	73	58	597,9
White Prat	11/16/2016	88	81	78	66	960,6
Blue Andaluza	6/20/2016	78		73		2602,4
Blue Andaluza	6/27/2016	85		60		1899,0
Blue Andaluza	7/4/2016	68		64		2373,7
Blue Andaluza	7/13/2016	79		74		3505,0
Blue Andaluza	7/18/2016	77	84	100	51	483,7
Blue Andaluza	8/10/2016	74	80	52	52	1659,4
Blue Andaluza	9/7/2016	82	67	48	55	2096,0
Blue Andaluza	9/19/2016	62	68	51	37	3620,9
Blue Andaluza	9/28/2016	87	61	65	65	1812,0
Blue Andaluza	10/10/2016	82	81	68	39	2042,6
Blue Andaluza	10/17/2016	89	68	47	35	1208,0
Blue Andaluza	10/26/2016	66	78	54	51	834,7
Blue Andaluza	11/10/2016	75	70	70	59	967,7
Blue Andaluza	11/16/2016	80	79	49	70	1098,7
Quail Silver Castelllana	6/27/2016	70		75		4962,4
Quail Silver Castelllana	7/4/2016	84		97		1576,5
Quail Silver Castelllana	7/13/2016	95		79		954
Quail Silver Castelllana	7/18/2016	85	59	40	71	1296,6
Quail Silver Castelllana	8/17/2016	95	82	26	58	1417,5
Quail Silver Castelllana	8/31/2016	81	75	67	52	1464,5
Quail Silver Castelllana	9/12/2016	62	68	67	37	1848,4
Quail Silver Castelllana	9/28/2016	84	77	45	30	1308,1
Quail Silver Castelllana	10/10/2016	69	87	70	40	1655

Quail Silver Castelllana	10/24/2016	75	67	70	46	1094,9
Quail Silver Castelllana	11/7/2016	69	52	64	44	833,1
Quail Silver Castelllana	11/10/2016	70	60	55	55	735,3
Quail Silver Castelllana	11/14/2016	83	57	55	47	559,1
Red Villafranquina	6/20/2016	71				4377,3
Red Villafranquina	6/27/2016	75		76		4317,4
Red Villafranquina	7/4/2016	79		64		3878,5
Red Villafranquina	7/13/2016	52		70		1083,8
Red Villafranquina	7/27/2016	99	63	48	43	6010,4
Red Villafranquina	8/17/2016	88	73	55	34	3500
Red Villafranquina	8/31/2016	60	63	59	46	2096
Red Villafranquina	9/12/2016	84	51	52	55	3494,3
Red Villafranquina	9/28/2016	78	65	65	53	2494,9
Red Villafranquina	10/10/2016	86	80	48	18	2010,2
Red Villafranquina	10/24/2016	86	70	33	55	1632,9
Red Villafranquina	11/7/2016	87	47	69	48	646,5
Red Villafranquina	11/14/2016	83	61	62	49	486,2
Red Villafranquina	11/21/2016	95	55	79	54	868,9
Black-Barred Andaluza	6/20/2016	72				2950
Black-Barred Andaluza	6/27/2016	78		77		2732
Black-Barred Andaluza	7/4/2016	81				4048,7
Black-Barred Andaluza	7/13/2016	89		50		430
Black-Barred Andaluza	7/27/2016	98	66	46	74	931,6
Black-Barred Andaluza	8/17/2016	79	78	35	44	2579,7
Black-Barred Andaluza	8/31/2016	67	69	41	54	2310,3
Black-Barred Andaluza	9/12/2016	83	78	58		1733,1
Black-Barred Andaluza	9/21/2016	66	84	33	33	3279,8
Black-Barred Andaluza	10/3/2016	84	86	47	48	2491,8
Black-Barred Andaluza	10/24/2016	60	55	32	25	1286,9
Black-Barred Andaluza	11/7/2016	87	47	59	57	588,8
Black-Barred Andaluza	11/14/2016	81	82	59	62	1241,4
Black-Barred Andaluza	11/21/2016	93	44	60	32	606,4
White-Faced Spanish	6/20/2016	74				2248,6

White-Faced Spanish	6/27/2016	79		65		164,3
White-Faced Spanish	7/4/2016			51		2006,4
White-Faced Spanish	7/13/2016	90		32		1397,3
White-Faced Spanish	7/27/2016	67	56	32	48	1737,7
White-Faced Spanish	8/17/2016	76	70	54	48	2472,2
White-Faced Spanish	8/31/2016	78	70	57	44	2101
White-Faced Spanish	9/12/2016	81	48	51	44	1763,4
White-Faced Spanish	9/21/2016	74	70	51	44	2220,6
White-Faced Spanish	10/3/2016	75	73	56	45	3513,8
White-Faced Spanish	10/24/2016	64	34	57	56	937,9
White-Faced Spanish	11/7/2016	93	62	61	49	714,8
White-Faced Spanish	11/14/2016	92	61	56	45	383,9
White-Faced Spanish	11/21/2016	87	62	53	65	236,7
Black Castellana	6/20/2016	64				3327,9
Black Castellana	6/27/2016	83		70		2615,5
Black Castellana	7/4/2016	84		87		1576,5
Black Castellana	7/13/2016	91				994,3
Black Castellana	7/27/2016	82	48	63	33	2857,4
Black Castellana	8/17/2016	91	86	73	55	2561,8
Black Castellana	8/31/2016	72	76	63	52	1076,1
Black Castellana	9/12/2016	90	69	49	39	1906,1
Black Castellana	9/28/2016	61	62	45	52	2722,5
Black Castellana	10/10/2016	88	78	40	46	1731,4
Black Castellana	10/24/2016	68	77	59	67	828,6
Black Castellana	11/7/2016	70	54	59	43	1132,1
Black Castellana	11/16/2016	95	58	64	73	742,1
Black Castellana	11/21/2016	74	48	77	48	525,1
Buff Prat	6/20/2016	69				3543,8
Buff Prat	6/27/2016	60		75		3780
Buff Prat	7/4/2016	72				3815,8
Buff Prat	7/13/2016	89		78		3672,5
Buff Prat	7/27/2016	85		41	43	2382,5
Buff Prat	8/17/2016	88	74	42	35	2009,3

Buff Prat	8/31/2016	75	65	66	46	3559
Buff Prat	9/12/2016	88	87	65	37	1605,6
Buff Prat	9/28/2016	79	64	61	45	2164,1
Buff Prat	10/10/2016	71	70	44	43	1966,8
Buff Prat	10/24/2016	89	69	40	59	2998,7
Buff Prat	11/7/2016	94	79	73	70	560
Buff Prat	11/14/2016	90	71	66	51	814,9
Buff Prat	11/21/2016	92	67	80	67	1104,8
