# Supplementary material for: Seminal plasma amino acid profile in different breeds of chicken: Role of seminal plasma on sperm cryoresistance
Source: PLoS One. 2019 Jan 4;14(1):e0209910. doi: 10.1371/journal.pone.0209910 (PMC6319765; doi:10.1371/journal.pone.0209910)
Supplement: S4 Dataset — (PDF) [file pone.0209910.s004.pdf]

**S4 Dataset. Motility variables of sperm of 12 Spanish rooster breeds (fresh, frozen with plasma and frozen without plasma).**

**Rooster B Treatment**

| Rooster Breed | Treatment |
|---|---|
| 1 = Blue Andaluza | 1 = Fresh semen |
| 2 = Black-Red Andaluza | 2 = Frozen with plasma (w/p) |
| 3 = White-Faced Spanish | 3 = Frozen without (w/o/p) |
| 4= Quail Castellana | |
| 5 = Black Castellana | |
| 6 = Quail Silver Castellana | |
| 7 = Black-Barred Andaluza | |
| 8 = Birchen Leonesa | |
| 9 = White Prat | |
| 10 = Buff Prat | |
| 11= Red-Barred Vasca | |
| 12 = Red Villafranquina | |

| Rooster Breed | Treatment | Date | Motility % | score | Statics % | No progressive motiles % | Progressive Motiles % | Total Motiles % | VCL µm/s | VSL µm/s | VAP µm/s | LIN % | STR % | WOB % | ALH µm | BCF Hz |
|---|---|---|---|---|---|---|---|---|---|---|---|---|---|---|---|---|
| 1 | 1 | 6/20/2016 | 70,0 | 2,5 | 30,6 | 51,5 | 17,8 | 69,3 | 41,3 | 20,2 | 27,4 | 49,0 | 73,7 | 66,4 | 2,7 | 8,5 |
| 1 | 1 | 6/27/2016 | 90,0 | 3,5 | 11,4 | 28,5 | 6,0 | 34,5 | 71,9 | 51,0 | 57,4 | 70,9 | 88,8 | 79,9 | 2,6 | 10,0 |
| 1 | 1 | 7/4/2016 | 20,0 | 1,0 | 99,2 | 0,8 | 0,0 | 0,8 | 9,8 | 0,4 | 2,8 | 4,0 | 14,1 | 28,0 | 0,0 | 0,0 |
| 1 | 1 | 7/13/2016 | 75,0 | 2,5 | 24,0 | 31,3 | 44,7 | 76,0 | 82,3 | 48,5 | 60,8 | 59,0 | 79,8 | 73,9 | 3,7 | 8,5 |
| 1 | 1 | 7/18/2016 | 95,0 | 3,0 | 20,7 | 37,9 | 41,4 | 79,3 | 38,7 | 23,3 | 31,3 | 60,3 | 74,6 | 80,8 | 1,4 | 4,1 |
| 1 | 1 | 8/10/2016 | 90,0 | 4,0 | 36,0 | 25,7 | 38,3 | 64,0 | 74,9 | 53,8 | 61,8 | 71,8 | 87,0 | 82,5 | 2,8 | 9,3 |
| 1 | 1 | 9/7/2016 | 80,0 | 4,0 | 9,3 | 21,1 | 69,5 | 90,6 | 111,1 | 87,3 | 97,8 | 78,5 | 89,3 | 88,0 | 2,5 | 9,2 |
| 1 | 1 | 9/19/2016 | 75,0 | 2,5 | 54,4 | 38,9 | 6,6 | 45,5 | 37,5 | 15,3 | 23,8 | 40,7 | 64,2 | 63,4 | 3,3 | 7,8 |
| 1 | 1 | 9/28/2016 | 85,0 | 3,0 | 17,9 | 41,1 | 41,1 | 82,2 | 81,5 | 50,2 | 64,6 | 61,6 | 77,7 | 79,3 | 2,8 | 8,7 |
| 1 | 1 | 10/10/2016 | 70,0 | 2,5 | 43,9 | 39,1 | 17,0 | 56,1 | 49,7 | 23,9 | 32,9 | 48,1 | 72,7 | 66,2 | 3,1 | 9,2 |
| 1 | 1 | 10/17/2016 | 90,0 | 3,5 | 17,3 | 32,7 | 50,0 | 82,7 | 89,2 | 57,7 | 68,6 | 64,7 | 84,1 | 76,9 | 2,9 | 8,4 |
| 1 | 1 | 10/26/2016 | 90,0 | 3,5 | 14,2 | 35,3 | 50,5 | 85,8 | 100,7 | 64,7 | 81,7 | 64,2 | 79,2 | 81,1 | 3,3 | 9,6 |
| 1 | 1 | 11/10/2016 | 95,0 | 3,5 | 12,4 | 27,8 | 59,8 | 87,6 | 99,1 | 72,2 | 84,6 | 72,9 | 85,4 | 85,4 | 2,8 | 9,3 |
| 1 | 1 | 11/16/2016 | 85,0 | 3,5 | 14,0 | 24,6 | 61,4 | 86,0 | 97,9 | 70,1 | 81,6 | 71,6 | 85,9 | 83,3 | 3,1 | 9,8 |

| | | | | | | | | | | | | | | | | |
|---|---|---|---|---|---|---|---|---|---|---|---|---|---|---|---|---|
| 1 | 2 | 6/20/2016 | 75,0 | 3,0 | 64,0 | 33,0 | 3,0 | 36,0 | 30,7 | 9,9 | 17,2 | 32,2 | 57,3 | 56,2 | 3,0 | 6,9 |
| 1 | 2 | 6/27/2016 | 60,0 | 3,5 | 72,3 | 24,1 | 3,6 | 27,7 | 31,8 | 16,3 | 22,4 | 51,3 | 72,9 | 70,3 | 2,9 | 9,7 |
| 1 | 2 | 7/13/2016 | 90,0 | 3,0 | 17,6 | 54,9 | 27,9 | 82,8 | 60,3 | 28,2 | 39,3 | 46,8 | 71,8 | 65,1 | 3,0 | 8,8 |
| 1 | 2 | 7/18/2016 | 20,0 | 1,5 | | | | | | | | | | | | |
| 1 | 2 | 8/10/2016 | 80,0 | 2,5 | 42,9 | 39,5 | 17,6 | 57,1 | 51,4 | 27,2 | 35,8 | 52,8 | 75,9 | 69,5 | 2,9 | 9,3 |
| 1 | 2 | 9/7/2016 | 40,0 | 2,5 | 71,2 | 26,4 | 2,4 | 28,8 | 35,2 | 11,3 | 18,4 | 32,1 | 61,4 | 52,3 | 3,1 | 7,1 |
| 1 | 2 | 9/19/2016 | 70,0 | 3,0 | 42,0 | 32,7 | 25,3 | 58,0 | 64,0 | 42,1 | 50,0 | 65,8 | 84,2 | 78,2 | 2,6 | 9,9 |
| 1 | 2 | 9/28/2016 | 60,0 | 2,0 | 27,8 | 47,3 | 24,9 | 72,2 | 56,0 | 28,7 | 38,1 | 51,4 | 75,5 | 68,0 | 2,8 | 8,9 |
| 1 | 2 | 10/10/2016 | 50,0 | 3,0 | 78,6 | 14,1 | 7,3 | 21,4 | 52,5 | 40,1 | 45,4 | 76,4 | 88,3 | 86,6 | 2,0 | 8,6 |
| 1 | 2 | 10/17/2016 | 30,0 | 1,0 | 85,4 | 10,2 | 4,4 | 14,6 | 47,0 | 22,1 | 29,6 | 47,1 | 74,8 | 63,0 | 2,8 | 9,0 |
| 1 | 2 | 10/26/2016 | 70,0 | 2,0 | 69,7 | 17,9 | 12,4 | 30,3 | 63,2 | 41,1 | 48,6 | 65,0 | 84,5 | 77,0 | 2,4 | 10,3 |
| 1 | 2 | 11/10/2016 | 40,0 | 3,0 | 62,1 | 15,9 | 22,0 | 37,9 | 75,1 | 52,9 | 59,2 | 70,5 | 89,3 | 78,9 | 2,9 | 10,5 |
| 1 | 2 | 11/16/2016 | 45,0 | 3,0 | 80,7 | 9,5 | 9,8 | 19,3 | 65,0 | 47,2 | 51,8 | 72,6 | 91,2 | 79,6 | 2,7 | 8,8 |
| 1 | 3 | 7/18/2016 | 40,0 | 1,5 | 55,3 | 37,3 | 7,5 | 44,8 | 37,5 | 16,8 | 24,0 | 44,9 | 70,3 | 63,9 | 2,9 | 8,7 |
| 1 | 3 | 8/10/2016 | 75,0 | 2,0 | 30,9 | 52,4 | 16,7 | 69,1 | 48,4 | 21,8 | 31,9 | 45,1 | 68,5 | 65,8 | 2,8 | 8,4 |
| 1 | 3 | 9/7/2016 | 75,0 | 2,0 | 26,3 | 57,0 | 16,7 | 73,7 | 43,7 | 19,0 | 27,9 | 43,4 | 68,1 | 63,7 | 2,7 | 7,8 |
| 1 | 3 | 9/19/2016 | 40,0 | 2,0 | 74,3 | 21,6 | 4,1 | 25,7 | 35,6 | 15,8 | 22,7 | 44,5 | 69,9 | 63,7 | 2,7 | 8,1 |
| 1 | 3 | 9/28/2016 | 30,0 | 1,0 | 66,5 | 31,0 | 2,5 | 33,5 | 41,4 | 10,9 | 21,0 | 26,3 | 51,9 | 50,8 | 3,4 | 7,5 |
| 1 | 3 | 10/10/2016 | 50,0 | 2,5 | 70,3 | 21,3 | 8,4 | 29,7 | 48,8 | 26,7 | 34,4 | 54,7 | 77,5 | 70,6 | 2,5 | 5,8 |
| 1 | 3 | 10/17/2016 | 25,0 | 1,5 | 79,3 | 15,5 | 5,2 | 20,7 | 48,3 | 24,4 | 30,5 | 50,6 | 80,1 | 63,1 | 2,2 | 8,3 |
| 1 | 3 | 10/26/2016 | 40,0 | 2,5 | 68,3 | 22,2 | 9,5 | 31,7 | 54,8 | 28,3 | 37,4 | 51,7 | 75,7 | 68,2 | 2,9 | 9,0 |
| 1 | 3 | 11/10/2016 | 35,0 | 2,0 | 79,4 | 14,0 | 6,5 | 20,5 | 53,5 | 32,4 | 37,9 | 60,7 | 85,6 | 70,9 | 2,1 | 9,6 |
| 1 | 3 | 11/16/2016 | 40,0 | 3,0 | 80,7 | 9,5 | 9,8 | 19,3 | 65,0 | 47,2 | 51,8 | 72,6 | 91,2 | 79,6 | 2,7 | 8,8 |
| 2 | 1 | 6/20/2016 | 75 | 3,5 | 3,9 | 39,4 | 56,7 | 96,1 | 107,3 | 67,8 | 86,6 | 63,2 | 78,2 | 80,7 | 3,3 | 8,7 |
| 2 | 1 | 6/27/2016 | 80 | 3 | 24,4 | 51,1 | 24,4 | 75,5 | 51,1 | 32,7 | 39,2 | 64,0 | 83,3 | 76,8 | 2,7 | 9,7 |
| 2 | 1 | 7/4/2016 | 15 | 1 | 91,1 | 8,9 | 0 | 8,9 | 20,2 | 8,0 | 12,4 | 39,5 | 64,5 | 61,2 | 0,0 | 0,0 |
| 2 | 1 | 7/13/2016 | | | 60,9 | 29,1 | 10 | 39,1 | 44,2 | 23,9 | 30,0 | 54,0 | 79,7 | 67,8 | 2,8 | 10,1 |
| 2 | 1 | 7/18/2016 | 90 | 3 | 28,8 | 36,2 | 35 | 71,2 | 77,5 | 46,5 | 59,2 | 60,1 | 78,6 | 76,5 | 3,1 | 9,2 |
| 2 | 1 | 8/10/2016 | 50 | 3 | 37,1 | 32,6 | 30,3 | 62,9 | 65,5 | 43,5 | 52,2 | 66,4 | 83,3 | 79,7 | 2,8 | 9,7 |
| 2 | 1 | 9/7/2016 | 75 | 3 | 16,1 | 30,7 | 53,2 | 83,9 | 77,9 | 49,7 | 61,0 | 63,8 | 81,5 | 78,2 | 3,1 | 9,1 |
| 2 | 1 | 9/19/2016 | 75 | 2 | 57,7 | 37,7 | 4,6 | 42,3 | 34,2 | 11,0 | 19,7 | 32,2 | 55,9 | 57,7 | 3,2 | 7,6 |
| 2 | 1 | 9/28/2016 | 90 | 2 | 11,5 | 47,7 | 40,8 | 88,5 | 69,3 | 35,4 | 49,3 | 51,0 | 71,8 | 71,0 | 3,2 | 7,7 |
| 2 | 1 | 10/10/2016 | 85 | 3 | 9,6 | 43,8 | 46,6 | 90,4 | 89,9 | 52,8 | 69,2 | 58,7 | 76,3 | 77,0 | 3,5 | 8,7 |

| | | | | | | | | | | | | | | | | |
|---|---|---|---|---|---|---|---|---|---|---|---|---|---|---|---|---|
| 2 | 1 | 10/17/2016 | 90 | 3,5 | 15 | 40,3 | 44,7 | 85 | 99,9 | 61,8 | 80,1 | 61,8 | 77,1 | 80,1 | 3,3 | 8,4 |
| 2 | 1 | 10/26/2016 | 90 | 3 | 6,5 | 31,2 | 62,3 | 93,5 | 94,9 | 62,4 | 77,9 | 65,7 | 80,1 | 82,1 | 3,1 | 8,8 |
| 2 | 1 | 11/10/2016 | 90 | 3 | 20,6 | 25,8 | 53,6 | 79,4 | 81,0 | 58,8 | 67,7 | 72,6 | 86,8 | 83,6 | 2,7 | 8,9 |
| 2 | 1 | 11/16/2016 | 85 | 3 | 11,5 | 32,9 | 55,6 | 88,5 | 79,7 | 48,4 | 61,8 | 60,7 | 78,3 | 77,6 | 3,0 | 8,4 |
| 2 | 2 | 6/20/2016 | | | 77,6 | 21,2 | 1,2 | 22,4 | 25,8 | 8,3 | 14,0 | 32,3 | 59,7 | 54,1 | 2,5 | 8,5 |
| 2 | 2 | 6/27/2016 | 40 | 1,5 | 57 | 38,2 | 4,8 | 43 | 32,0 | 12,9 | 20,1 | 40,3 | 64,3 | 62,7 | 2,7 | 7,5 |
| 2 | 2 | 7/4/2016 | 40 | 2 | 81,6 | 14,2 | 4,2 | 18,4 | 50,4 | 21,6 | 31,9 | 42,8 | 67,6 | 63,3 | 2,8 | 6,8 |
| 2 | 2 | 7/13/2016 | 90 | 2,5 | 37,2 | 52,5 | 10,3 | 62,8 | 44,9 | 17,3 | 28,1 | 38,5 | 61,4 | 62,7 | 3,2 | 6,7 |
| 2 | 2 | 7/18/2016 | 50 | 1,5 | | | | | | | | | | | | |
| 2 | 2 | 8/10/2016 | 85 | 2,5 | 24,9 | 55,8 | 19,3 | 75,1 | 56,7 | 23,0 | 35,3 | 40,6 | 65,2 | 62,3 | 3,2 | 7,6 |
| 2 | 2 | 9/7/2016 | 40 | 1,5 | 74,8 | 24,1 | 1 | 25,1 | 24,6 | 10,0 | 15,4 | 40,7 | 65,0 | 62,7 | 2,4 | 6,8 |
| 2 | 2 | 9/19/2016 | 30 | 1 | 67,5 | 25,7 | 6,7 | 32,4 | 43,1 | 22,6 | 29,3 | 52,5 | 77,1 | 68,1 | 2,7 | 9,1 |
| 2 | 2 | 9/28/2016 | 40 | 1 | 60,2 | 35,9 | 3,9 | 39,8 | 35,3 | 13,3 | 21,5 | 37,6 | 61,6 | 61,0 | 3,0 | 9,3 |
| 2 | 2 | 10/10/2016 | 40 | 1,5 | 78,5 | 16 | 5,5 | 21,5 | 43,5 | 21,2 | 29,8 | 48,6 | 71,1 | 68,4 | 3,0 | 8,7 |
| 2 | 2 | 10/17/2016 | 45 | 2 | 85,7 | 8,4 | 5,9 | 14,3 | 70,0 | 46,5 | 57,1 | 66,4 | 81,3 | 81,7 | 2,8 | 7,0 |
| 2 | 2 | 10/26/2016 | 35 | 1,5 | 79,7 | 13,6 | 6,7 | 20,3 | 49,2 | 31,3 | 38,9 | 63,7 | 80,5 | 79,1 | 2,3 | 9,0 |
| 2 | 2 | 11/10/2016 | 15 | 1 | 84,9 | 11,1 | 4 | 15,1 | 55,4 | 32,1 | 39,1 | 58,0 | 82,1 | 70,6 | 2,3 | 8,6 |
| 2 | 2 | 11/16/2016 | 40 | 2 | 87 | 9,6 | 3,4 | 13 | 53,0 | 29,1 | 37,9 | 54,8 | 76,8 | 71,4 | 2,6 | 9,9 |
| 2 | 3 | 7/18/2016 | 20 | 1,5 | 88,2 | 8,4 | 3,4 | 11,8 | 46,9 | 33,9 | 37,5 | 72,3 | 90,5 | 79,9 | 1,8 | 7,5 |
| 2 | 3 | 8/10/2016 | 60 | 1,5 | 37,4 | 56,7 | 5,9 | 62,6 | 40,1 | 12,9 | 22,8 | 32,1 | 56,5 | 56,9 | 3,4 | 6,4 |
| 2 | 3 | 9/7/2016 | 40 | 1 | 61,4 | 35,6 | 2,9 | 38,5 | 30,3 | 10,9 | 18,2 | 35,9 | 60,0 | 59,9 | 2,7 | 8,8 |
| 2 | 3 | 9/19/2016 | 40 | 1,5 | 75,5 | 21,7 | 2,8 | 24,5 | 35,3 | 13,7 | 21,8 | 38,8 | 62,7 | 61,9 | 2,5 | 8,0 |
| 2 | 3 | 9/28/2016 | 75 | 2 | 25,2 | 50,2 | 24,6 | 74,8 | 55,0 | 27,2 | 39,2 | 49,6 | 69,4 | 71,4 | 3,0 | 7,8 |
| 2 | 3 | 10/10/2016 | 40 | 1 | 84,0 | 11,8 | 4,1 | 15,9 | 45,4 | 25,2 | 31,6 | 55,4 | 79,5 | 69,7 | 2,2 | 8,2 |
| 2 | 3 | 10/17/2016 | 40 | 2 | 67,5 | 19,4 | 13,1 | 32,5 | 64,3 | 36,8 | 49,5 | 57,2 | 74,3 | 76,9 | 2,9 | 9,4 |
| 2 | 3 | 10/26/2016 | 40 | 2,5 | 82,7 | 14,3 | 3,0 | 17,3 | 50,0 | 33,9 | 39,1 | 67,7 | 86,7 | 78,1 | 1,6 | 8,2 |
| 2 | 3 | 11/10/2016 | 20 | 1,5 | 85,3 | 11,9 | 2,8 | 14,7 | 44,6 | 21,3 | 28,5 | 47,7 | 74,5 | 64,0 | 1,5 | 5,8 |
| 2 | 3 | 11/16/2016 | 65 | 3 | 39,9 | 43,6 | 16,5 | 60,1 | 48,4 | 29,0 | 36,3 | 59,8 | 79,8 | 75,0 | 2,5 | 9,2 |
| 3 | 1 | 6/20/2016 | 40 | 2 | 36,3 | 59,6 | 4,2 | 63,8 | 27,7 | 12,2 | 18,3 | 44,1 | 66,6 | 66,2 | 2,8 | 9,7 |
| 3 | 1 | 6/27/2016 | 30 | 2 | 69,5 | 28,9 | 1,6 | 30,5 | 26,1 | 12,4 | 16,9 | 47,3 | 73,0 | 64,8 | 1,9 | 5,7 |
| 3 | 1 | 7/4/2016 | | | 82,4 | 17,1 | 0,5 | 17,6 | 18,1 | 7,2 | 10,7 | 39,8 | 67,1 | 59,2 | 1,0 | 4,3 |
| 3 | 1 | 7/13/2016 | 90 | 2,5 | 55,4 | 39,6 | 5 | 44,6 | 36,4 | 15,9 | 23,6 | 43,6 | 67,2 | 64,9 | 2,7 | 8,8 |
| 3 | 1 | 7/27/2016 | 70 | 1,5 | 66 | 30,4 | 3,7 | 34,1 | 26,5 | 13,2 | 18,2 | 50,0 | 72,5 | 68,9 | 1,7 | 4,3 |

| | | | | | | | | | | | | | | | | |
|---|---|---|---|---|---|---|---|---|---|---|---|---|---|---|---|---|
| 3 | 1 | 8/17/2016 | 76 | | 35 | 37,1 | 27,9 | 65 | 57,7 | 36,0 | 44,3 | 62,4 | 81,4 | 76,6 | 2,9 | 9,6 |
| 3 | 1 | 8/31/2016 | 80 | 2 | 43,8 | 37,1 | 19,1 | 56,2 | 51,2 | 28,7 | 36,1 | 56,0 | 79,5 | 70,5 | 2,9 | 9,3 |
| 3 | 1 | 9/12/2016 | 80 | 1,5 | 57,9 | 29,6 | 12,6 | 42,2 | 50,0 | 24,4 | 33,5 | 48,8 | 72,9 | 67,0 | 3,1 | 8,1 |
| 3 | 1 | 9/21/2016 | 80 | 3 | 51,6 | 34,4 | 14,1 | 48,5 | 44,9 | 24,9 | 32,9 | 55,3 | 75,6 | 73,2 | 3,0 | 8,1 |
| 3 | 1 | 10/3/2016 | 80 | 3 | 8 | 48,8 | 43,2 | 92 | 72,8 | 40,9 | 54,3 | 56,2 | 75,2 | 74,7 | 3,1 | 8,3 |
| 3 | 1 | 10/24/2016 | 70 | 1,5 | 38,7 | 32,5 | 28,8 | 61,3 | 54,3 | 30,8 | 38,8 | 56,8 | 79,5 | 71,4 | 2,8 | 9,7 |
| 3 | 1 | 11/7/2016 | 95 | 3,5 | 8,7 | 31,2 | 60,1 | 91,3 | 82,5 | 51,0 | 64,5 | 61,8 | 79,0 | 78,2 | 3,2 | 8,9 |
| 3 | 1 | 11/14/2016 | 80 | 2 | 16,7 | 31,6 | 51,6 | 83,2 | 81,0 | 56,8 | 66,3 | 70,1 | 85,6 | 81,9 | 3,0 | 9,4 |
| 3 | 1 | 11/21/2016 | 90 | 3,5 | 19,3 | 33,8 | 46,9 | 80,7 | 81,6 | 57,2 | 65,9 | 70,2 | 86,8 | 80,8 | 3,0 | 9,3 |
| 3 | 2 | 6/20/2016 | | | 59,5 | 36,4 | 4,1 | 40,5 | 31,8 | 10,9 | 17,8 | 34,1 | 61,0 | 55,9 | 2,9 | 9,6 |
| 3 | 2 | 6/27/2016 | 30 | 1 | 77,7 | 20,5 | 1,9 | 22,4 | 31,2 | 11,6 | 18,4 | 37,3 | 63,4 | 58,9 | 2,9 | 9,9 |
| 3 | 2 | 7/4/2016 | 20 | 1,5 | 77,9 | 19,5 | 2,6 | 22,1 | 29,3 | 12,2 | 17,6 | 41,5 | 69,2 | 59,9 | 3,2 | 9,1 |
| 3 | 2 | 7/13/2016 | 40 | 1 | 84 | 12,4 | 3,6 | 16 | 69,0 | 25,9 | 42,4 | 37,5 | 61,0 | 61,4 | 2,5 | 7,4 |
| 3 | 2 | 7/27/2016 | 80 | 2,5 | 41,5 | 45,2 | 13,2 | 58,4 | 43,1 | 20,8 | 29,9 | 48,3 | 69,6 | 69,4 | 2,8 | 7,4 |
| 3 | 2 | 8/17/2016 | 80 | 2,5 | 50,5 | 40,9 | 8,5 | 49,4 | 45,4 | 17,0 | 26,3 | 37,6 | 64,8 | 58,0 | 3,5 | 7,4 |
| 3 | 2 | 8/31/2016 | 80 | 2 | 34,6 | 47,2 | 18,2 | 65,4 | 51,1 | 24,9 | 34,7 | 48,8 | 71,8 | 68,0 | 3,0 | 8,1 |
| 3 | 2 | 9/12/2016 | 60 | 1,5 | | | | | | | | | | | | |
| 3 | 2 | 9/21/2016 | 80 | 2 | 29,9 | 57,8 | 12,3 | 70,1 | 44,5 | 19,7 | 29,2 | 44,3 | 67,5 | 65,7 | 2,7 | 8,4 |
| 3 | 2 | 10/3/2016 | 65 | 2 | | | | | | | | | | | | |
| 3 | 2 | 10/24/2016 | 40 | 2 | | | | | | | | | | | | |
| 3 | 2 | 11/7/2016 | 30 | 2 | 83,4 | 7,8 | 8,8 | 16,6 | 76,2 | 58,4 | 63,6 | 76,6 | 91,8 | 83,5 | 2,5 | 12,3 |
| 3 | 2 | 11/14/2016 | 30 | 2 | 82,5 | 8,4 | 9,1 | 17,5 | 60,7 | 43,3 | 47,9 | 71,2 | 90,2 | 79,0 | 2,3 | 7,2 |
| 3 | 2 | 11/21/2016 | 10 | 1 | 80,6 | 12,9 | 6,5 | 19,4 | 45,8 | 23,9 | 30,4 | 52,2 | 78,6 | 66,5 | 2,1 | 7,4 |
| 3 | 3 | 7/27/2016 | 35 | 1,5 | | | | | | | | | | | | |
| 3 | 3 | 8/17/2016 | 35 | 1,5 | 77,0 | 19,1 | 3,9 | 23,0 | 36,0 | 17,8 | 23,7 | 49,5 | 75,4 | 65,7 | 2,5 | 7,9 |
| 3 | 3 | 8/31/2016 | 30 | 2 | 48,8 | 41,9 | 9,3 | 51,2 | 37,9 | 17,2 | 24,9 | 45,5 | 69,2 | 65,7 | 2,8 | 8,9 |
| 3 | 3 | 9/12/2016 | 40 | 1,5 | 61,4 | 32,0 | 6,6 | 38,6 | 36,0 | 15,3 | 22,6 | 42,6 | 67,7 | 62,9 | 2,9 | 7,5 |
| 3 | 3 | 9/21/2016 | 50 | 1,5 | 68,5 | 26,4 | 5,1 | 31,5 | 39,3 | 17,5 | 25,1 | 44,6 | 69,7 | 64,0 | 2,8 | 8,9 |
| 3 | 3 | 10/3/2016 | 40 | 2 | 91,8 | 5,8 | 2,4 | 8,2 | 67 | 49,2 | 55,2 | 73,5 | 89,2 | 82,5 | 1,7 | 6,2 |
| 3 | 3 | 10/24/2016 | 30 | 3 | 88,0 | 7,7 | 4,3 | 12,0 | 54,0 | 26,4 | 33,7 | 49,0 | 78,6 | 62,3 | 2,4 | 4,1 |
| 3 | 3 | 11/7/2016 | 60 | 2,5 | 80,0 | 11,5 | 8,5 | 20,0 | 83,9 | 64,7 | 70,0 | 77,2 | 92,5 | 83,5 | 2,5 | 12,3 |
| 3 | 3 | 11/14/2016 | 20 | 1 | 88,6 | 7,8 | 3,6 | 11,4 | 49,1 | 31,4 | 35,5 | 63,9 | 88,5 | 72,3 | 1,6 | 3,9 |
| 3 | 3 | 11/21/2016 | 20 | 1,5 | 82,4 | 12,5 | 5,1 | 17,6 | 53,8 | 24,8 | 35,1 | 46,2 | 70,7 | 65,4 | 2,7 | 10,4 |

| | | | | | | | | | | | | | | | | |
|---|---|---|---|---|---|---|---|---|---|---|---|---|---|---|---|---|
| 4 | 1 | 6/20/2016 | 85 | 4 | 2,8 | 23 | 74,2 | 97,2 | 130,1 | 101,8 | 115,4 | 78,3 | 88,2 | 88,7 | 3,0 | 9,9 |
| 4 | 1 | 6/27/2016 | 75 | 3 | 25,9 | 47,1 | 27,1 | 74,2 | 53,0 | 34,0 | 39,8 | 64,1 | 85,4 | 75,0 | 2,8 | 9,9 |
| 4 | 1 | 7/4/2016 | 40 | 1,5 | 65 | 33,8 | 1,3 | 35,1 | 22,0 | 11,5 | 15,3 | 52,3 | 75,4 | 69,3 | 1,2 | 6,7 |
| 4 | 1 | 7/13/2016 | 80 | 2,5 | 22,2 | 28,4 | 49,4 | 77,8 | 86,5 | 52,4 | 66,7 | 60,6 | 78,6 | 77,2 | 3,1 | 8,8 |
| 4 | 1 | 7/18/2016 | 90 | 3 | | | | | | | | | | | | |
| 4 | 1 | 8/10/2016 | 90 | 4,5 | 11,6 | 27,5 | 61 | 88,5 | 123,9 | 86,9 | 104,5 | 70,1 | 83,1 | 84,4 | 3,2 | 8,9 |
| 4 | 1 | 9/7/2016 | 90 | 4 | 7,7 | 31,1 | 61,2 | 92,3 | 101,0 | 68,9 | 82,1 | 68,2 | 83,9 | 81,3 | 3,0 | 9,4 |
| 4 | 1 | 9/19/2016 | 90 | 2,5 | 48,9 | 41,3 | 9,8 | 51,1 | 41,3 | 16,2 | 25,0 | 39,2 | 64,8 | 60,4 | 3,3 | 8,5 |
| 4 | 1 | 9/21/2016 | 70 | 3,5 | 16,7 | 35,1 | 48,2 | 83,3 | 76,7 | 51,5 | 62,6 | 67,1 | 82,2 | 81,6 | 2,9 | 9,2 |
| 4 | 1 | 10/3/2016 | 80 | 2,5 | 11 | 51,1 | 38 | 89,1 | 74,8 | 37,2 | 52,5 | 49,7 | 70,8 | 70,2 | 3,2 | 8,4 |
| 4 | 1 | 10/17/2016 | 90 | 3 | 5,4 | 42,4 | 52,2 | 94,6 | 103,6 | 61,0 | 81,0 | 58,9 | 75,3 | 78,2 | 3,5 | 8,9 |
| 4 | 1 | 10/26/2016 | 40 | 1,5 | 60,2 | 37,3 | 2,6 | 39,9 | 27,9 | 11,3 | 16,8 | 40,3 | 66,8 | 60,4 | 2,7 | 9,8 |
| 4 | 1 | 11/10/2016 | 90 | 3 | 10 | 21,4 | 68,5 | 89,9 | 105,3 | 79,5 | 89,2 | 75,5 | 89,1 | 84,7 | 2,8 | 10,1 |
| 4 | 1 | 11/16/2016 | 90 | 3,5 | 12,9 | 36 | 51 | 87 | 79,6 | 47,3 | 59,7 | 59,4 | 79,2 | 75,1 | 3,1 | 9,3 |
| 4 | 2 | 6/27/2016 | 75 | 2 | 49,8 | 46,7 | 3,5 | 50,2 | 32,1 | 11,4 | 19,3 | 35,4 | 59,0 | 60,0 | 2,9 | 8,6 |
| 4 | 2 | 7/13/2016 | 70 | 1,5 | 75,6 | 21,9 | 2,5 | 24,4 | 33,4 | 12,9 | 20,3 | 38,8 | 63,9 | 60,7 | 4,3 | 9,2 |
| 4 | 2 | 7/18/2016 | 40 | 1 | 55,9 | 38,4 | 5,7 | 44,1 | 34,8 | 14,0 | 20,7 | 40,3 | 67,7 | 59,5 | 2,8 | 8,2 |
| 4 | 2 | 8/10/2016 | 30 | 1,5 | 92,1 | 6 | 1,9 | 7,9 | 73,6 | 59,8 | 64,9 | 81,3 | 92,2 | 88,2 | 2,5 | 6,3 |
| 4 | 2 | 9/7/2016 | 80 | 2 | 47,8 | 46,3 | 5,9 | 52,2 | 40,1 | 13,6 | 22,5 | 33,9 | 60,4 | 56,0 | 2,6 | 8,4 |
| 4 | 2 | 9/19/2016 | 70 | 1,5 | 46,9 | 43,6 | 9,5 | 53,1 | 44,4 | 16,6 | 26,3 | 37,5 | 63,2 | 59,4 | 3,0 | 7,0 |
| 4 | 2 | 9/21/2016 | 40 | 2,5 | 43,8 | 41,1 | 15,1 | 56,2 | 50,2 | 24,5 | 33,3 | 48,8 | 73,6 | 66,3 | 3,2 | 8,3 |
| 4 | 2 | 10/3/2016 | 40 | 1,5 | 83,1 | 14,9 | 2 | 16,9 | 43,5 | 21,8 | 26,9 | 50,0 | 80,9 | 61,9 | 3,6 | 10,4 |
| 4 | 2 | 10/17/2016 | 30 | 1 | 83,6 | 13,8 | 2,7 | 16,5 | 40,0 | 20,8 | 25,6 | 51,9 | 81,2 | 64,0 | 3,4 | 6,7 |
| 4 | 2 | 10/26/2016 | 60 | 1,5 | 87,4 | 7,3 | 5,3 | 12,6 | 58,8 | 39,4 | 46,6 | 67,0 | 84,6 | 79,3 | 2,2 | 6,8 |
| 4 | 2 | 11/10/2016 | 70 | 2,5 | 50 | 25,4 | 24,6 | 50 | 64,9 | 42,3 | 49,4 | 65,1 | 85,7 | 76,0 | 2,8 | 9,6 |
| 4 | 2 | 11/16/2016 | 60 | 2,5 | | | | | | | | | | | | |
| 4 | 3 | 7/18/2016 | 70 | 1,5 | 70,8 | 20,3 | 9 | 29,3 | 55,4 | 36,6 | 42,5 | 66,0 | 86,0 | 76,8 | 3,0 | 6,4 |
| 4 | 3 | 8/10/2016 | 15 | 1 | 87,8 | 10,1 | 2,1 | 12,2 | 42,1 | 23,4 | 30,0 | 55,6 | 78,0 | 71,3 | 2,2 | 9,5 |
| 4 | 3 | 9/7/2016 | 45 | 1 | 52,4 | 37,3 | 10,3 | 47,6 | 40,9 | 19,4 | 26,5 | 47,4 | 73,3 | 64,7 | 2,8 | 9,6 |
| 4 | 3 | 9/19/2016 | 40 | 1,5 | 44,6 | 43,6 | 11,8 | 55,4 | 47,8 | 19,4 | 29,2 | 40,5 | 66,2 | 61,2 | 3,0 | 7,3 |
| 4 | 3 | 9/21/2016 | 55 | 1,5 | 51,4 | 35,1 | 13,5 | 48,6 | 45,3 | 23,8 | 31,7 | 52,6 | 75,1 | 70,0 | 2,7 | 8,0 |
| 4 | 3 | 10/3/2016 | 30 | 1 | 90,8 | 8,4 | 0,8 | 9,2 | 36,5 | 17,4 | 21,9 | 47,6 | 79,4 | 60,0 | 1,4 | 7,8 |
| 4 | 3 | 10/17/2016 | 30 | 1,5 | 87,4 | 10,9 | 1,7 | 12,6 | 42,0 | 18,9 | 26,1 | 44,9 | 72,2 | 62,2 | 3,3 | 3,4 |

| | | | | | | | | | | | | | | | | |
|---|---|---|---|---|---|---|---|---|---|---|---|---|---|---|---|---|
| 4 | 3 | 10/26/2016 | 40 | 1 | 88,3 | 8,5 | 3,2 | 11,7 | 48,9 | 21,8 | 33,0 | 44,5 | 66,0 | 67,5 | 2,4 | 8,4 |
| 4 | 3 | 11/10/2016 | 15 | 1 | | | | | | | | | | | | |
| 4 | 3 | 11/16/2016 | 30 | 1,5 | 29,6 | 45,0 | 25,4 | 70,4 | 47,2 | 31,3 | 37,0 | 66,3 | 84,5 | 78,5 | 2,5 | 8,6 |
| 5 | 1 | 6/20/2016 | 65 | 4 | 13,8 | 42,9 | 43,3 | 86,2 | 73,2 | 48,4 | 58,8 | 66,1 | 82,2 | 80,4 | 2,9 | 9,0 |
| 5 | 1 | 6/27/2016 | 5 | 1,5 | 81,3 | 18,7 | 0 | 18,7 | 20,8 | 6,3 | 11,3 | 30,2 | 55,9 | 54,1 | 0,0 | 0,0 |
| 5 | 1 | 7/4/2016 | | | 21,6 | 29,4 | 49 | 78,4 | 82,2 | 58,1 | 67,3 | 70,6 | 86,3 | 81,8 | 2,9 | 9,8 |
| 5 | 1 | 7/13/2016 | 90 | 2 | 23,7 | 23,7 | 52,6 | 76,3 | 80,7 | 61,9 | 68,2 | 76,7 | 90,8 | 84,5 | 2,7 | 9,6 |
| 5 | 1 | 7/27/2016 | 50 | 1,5 | 59,9 | 34,4 | 5,7 | 40,1 | 33,2 | 17,6 | 23,2 | 53,0 | 75,9 | 69,8 | 2,5 | 9,3 |
| 5 | 1 | 8/17/2016 | 50 | 3 | 44,3 | 42,3 | 13,4 | 55,7 | 43,3 | 24,5 | 31,5 | 56,5 | 77,8 | 72,7 | 2,5 | 8,8 |
| 5 | 1 | 8/31/2016 | 90 | 2 | 8,8 | 17 | 74,2 | 91,2 | 94,3 | 75,7 | 81,4 | 80,3 | 93,0 | 86,3 | 2,7 | 11,2 |
| 5 | 1 | 9/12/2016 | 85 | 2 | 24,8 | 58,6 | 16,6 | 75,2 | 51,3 | 25,1 | 34,8 | 48,9 | 72,2 | 67,7 | 2,9 | 8,5 |
| 5 | 1 | 9/28/2016 | 75 | 2,5 | 50,1 | 43,1 | 6,9 | 50 | 35,9 | 14,4 | 22,8 | 40,0 | 63,1 | 63,4 | 3,1 | 9,2 |
| 5 | 1 | 10/10/2016 | 90 | 3,5 | 12,4 | 32,8 | 54,9 | 87,7 | 97,4 | 73,0 | 82,4 | 74,9 | 88,5 | 84,6 | 3,0 | 9,8 |
| 5 | 1 | 10/24/2016 | 40 | 1 | 69,3 | 19,3 | 11,5 | 30,8 | 40,7 | 21,7 | 27,9 | 53,4 | 77,9 | 68,5 | 2,4 | 9,2 |
| 5 | 1 | 11/7/2016 | 85 | 3 | 9,7 | 38,6 | 51,7 | 90,3 | 77,6 | 45,6 | 58,2 | 58,8 | 78,3 | 75,0 | 3,2 | 8,6 |
| 5 | 1 | 11/16/2016 | 85 | 2 | 8,8 | 30,7 | 60,6 | 91,3 | 84,8 | 56,4 | 66,1 | 66,5 | 85,3 | 77,9 | 3,4 | 9,3 |
| 5 | 1 | 11/21/2016 | 65 | 2 | 41,5 | 38,4 | 20,1 | 58,5 | 56,1 | 30,7 | 41,2 | 54,7 | 74,6 | 73,4 | 2,8 | 9,4 |
| 5 | 2 | 6/20/2016 | | | 54,8 | 36,4 | 8,9 | 45,3 | 39,9 | 16,3 | 24,5 | 40,8 | 66,4 | 61,5 | 3,1 | 7,5 |
| 5 | 2 | 6/27/2016 | 60 | 1,5 | 76,1 | 21,8 | 2,1 | 23,9 | 30,0 | 10,7 | 17,2 | 35,5 | 61,9 | 57,4 | 2,7 | 7,5 |
| 5 | 2 | 7/4/2016 | 10 | 1 | 92,3 | 6,7 | 1 | 7,7 | 29,6 | 10,8 | 17,8 | 36,4 | 60,6 | 60,2 | 1,8 | 6,1 |
| 5 | 2 | 7/13/2016 | 60 | 1,5 | 70,1 | 27,3 | 2,7 | 30 | 29,9 | 11,4 | 17,7 | 38,1 | 64,5 | 59,1 | 3,6 | 8,5 |
| 5 | 2 | 7/27/2016 | 40 | 1 | 91,3 | 6,1 | 2,5 | 8,6 | 49,8 | 32,0 | 39,2 | 64,3 | 81,7 | 78,7 | 2,4 | 7,5 |
| 5 | 2 | 8/17/2016 | 75 | 2 | 82,5 | 16,9 | 0,7 | 17,6 | 31,5 | 8,1 | 15,0 | 25,7 | 53,8 | 47,7 | 1,3 | 5,1 |
| 5 | 2 | 8/31/2016 | 30 | 1,5 | 72,4 | 24,3 | 3,2 | 27,5 | 35,5 | 15,4 | 22,2 | 43,4 | 69,6 | 62,4 | 3,2 | 7,8 |
| 5 | 2 | 9/12/2016 | 30 | 1,5 | | | | | | | | | | | | |
| 5 | 2 | 9/28/2016 | 75 | 2,5 | 19,2 | 69,1 | 11,8 | 80,9 | 40,6 | 17,0 | 25,7 | 41,8 | 66,0 | 63,3 | 3,1 | 7,8 |
| 5 | 2 | 10/10/2016 | 40 | 3 | 85,5 | 8,2 | 6,3 | 14,5 | 75,0 | 53,4 | 59,3 | 71,2 | 90,0 | 79,1 | 3,0 | 9,0 |
| 5 | 2 | 10/24/2016 | 40 | 1,5 | 70,8 | 20,6 | 8,6 | 29,2 | 59,6 | 30,3 | 39,7 | 50,8 | 76,3 | 66,6 | 3,9 | 7,9 |
| 5 | 2 | 11/7/2016 | 35 | 2 | 79,5 | 11,6 | 9 | 20,6 | 64,6 | 48,7 | 53,7 | 75,4 | 90,7 | 83,1 | 2,6 | 10,0 |
| 5 | 2 | 11/16/2016 | 40 | 2,5 | | | | | | | | | | | | |
| 5 | 2 | 11/21/2016 | 5 | 1 | | | | | | | | | | | | |
| 5 | 3 | 7/27/2016 | 30 | 1 | | | | | | | | | | | | |
| 5 | 3 | 8/17/2016 | 40 | 2 | 38,6 | 44,9 | 16,5 | 61,4 | 54,1 | 24,9 | 35,3 | 46,1 | 70,6 | 65,3 | 3,1 | 8,0 |

| | | | | | | | | | | | | | | | | |
|---|---|---|---|---|---|---|---|---|---|---|---|---|---|---|---|---|
| 5 | 3 | 8/31/2016 | 40 | 2 | 49,3 | 34,3 | 16,4 | 50,7 | 44,5 | 23,0 | 30,2 | 51,6 | 76,0 | 67,9 | 3,0 | 10,2 |
| 5 | 3 | 9/12/2016 | 40 | 1 | | | | | | | | | | | | |
| 5 | 3 | 9/28/2016 | 75 | 2 | 44,9 | 37,1 | 18,0 | 55,1 | 49,1 | 27,3 | 34,8 | 55,6 | 78,6 | 70,8 | 2,9 | 8,8 |
| 5 | 3 | 10/10/2016 | 40 | 1 | 79,7 | 16,1 | 4,2 | 20,3 | 43,8 | 21,1 | 27,9 | 48,1 | 75,5 | 63,7 | 2,6 | 8,2 |
| 5 | 3 | 10/24/2016 | 40 | 2 | 84,7 | 8,1 | 7,3 | 15,4 | 64,2 | 42,7 | 50,3 | 66,6 | 84,9 | 78,4 | 2,2 | 7,6 |
| 5 | 3 | 11/7/2016 | 35 | 1 | 78,6 | 15,9 | 5,5 | 21,4 | 47,9 | 21,1 | 29,6 | 44,1 | 71,4 | 61,7 | 3,0 | 10,4 |
| 5 | 3 | 11/16/2016 | 40 | 3 | 61,6 | 25,2 | 13,2 | 38,4 | 52,7 | 36,1 | 41,6 | 68,5 | 86,9 | 78,8 | 2,1 | 9,3 |
| 5 | 3 | 11/21/2016 | 35 | 2 | 78,2 | 14,8 | 7,0 | 21,8 | 50,5 | 29,5 | 37,3 | 58,5 | 79,2 | 73,8 | 2,1 | 7,9 |
| 6 | 1 | 6/27/2016 | 60 | 2,5 | 29,4 | 60,7 | 9,8 | 70,5 | 35,5 | 17,3 | 24,4 | 48,9 | 71,2 | 68,7 | 2,5 | 8,1 |
| 6 | 1 | 7/4/2016 | | | 58,8 | 39 | 2,2 | 41,2 | 27,8 | 11,2 | 18,2 | 40,1 | 61,1 | 65,7 | 3,2 | 8,0 |
| 6 | 1 | 7/13/2016 | 95 | 3 | 42,5 | 25,2 | 32,3 | 57,5 | 55,0 | 34,8 | 40,9 | 63,2 | 85,1 | 74,3 | 2,8 | 9,7 |
| 6 | 1 | 7/18/2016 | 90 | 3 | 8,6 | 11,2 | 80,2 | 91,4 | 112,0 | 96,1 | 101,6 | 85,8 | 94,6 | 90,7 | 2,4 | 10,3 |
| 6 | 1 | 8/17/2016 | 50 | 2 | 44,2 | 41,5 | 14,3 | 55,8 | 39,5 | 21,1 | 27,8 | 53,5 | 75,9 | 70,4 | 2,9 | 10,2 |
| 6 | 1 | 8/31/2016 | 90 | 3,5 | 19,9 | 31,4 | 48,7 | 80,1 | 69,8 | 49,4 | 55,8 | 70,7 | 88,5 | 79,9 | 2,9 | 10,5 |
| 6 | 1 | 9/12/2016 | 90 | 2,5 | 14,4 | 39,4 | 46,2 | 85,6 | 78,5 | 47,8 | 59,0 | 60,9 | 81,0 | 75,1 | 3,1 | 9,7 |
| 6 | 1 | 9/28/2016 | 95 | 3 | 23,9 | 47,6 | 28,4 | 76 | 57,4 | 31,9 | 41,6 | 55,5 | 76,8 | 72,3 | 3,3 | 8,4 |
| 6 | 1 | 10/10/2016 | 85 | 2 | 37,6 | 47,3 | 15,1 | 62,4 | 47,7 | 25,4 | 34,4 | 53,1 | 73,7 | 72,1 | 3,2 | 8,3 |
| 6 | 1 | 10/24/2016 | 85 | 2,5 | 32,5 | 34,4 | 33,1 | 67,5 | 59,9 | 32,6 | 40,4 | 54,5 | 80,7 | 67,5 | 2,8 | 8,8 |
| 6 | 1 | 11/7/2016 | 70 | 2 | 29,3 | 49,2 | 21,5 | 70,7 | 48,8 | 25,1 | 33,1 | 51,5 | 75,8 | 67,9 | 3,1 | 9,3 |
| 6 | 1 | 11/10/2016 | 80 | 2,5 | 18,1 | 41,7 | 40,2 | 81,9 | 71,9 | 47,1 | 57,1 | 65,4 | 82,4 | 79,4 | 2,9 | 8,6 |
| 6 | 1 | 11/14/2016 | 85 | 2,5 | 17,9 | 25,7 | 56,4 | 82,1 | 73,4 | 48,3 | 56,0 | 65,7 | 86,2 | 76,2 | 3,1 | 9,6 |
| 6 | 2 | 6/27/2016 | 60 | 2 | 62,6 | 32,6 | 4,8 | 37,4 | 35,0 | 11,3 | 18,4 | 32,5 | 61,7 | 52,6 | 3,1 | 8,5 |
| 6 | 2 | 7/4/2016 | 70 | 1,5 | 55,2 | 28,3 | 16,5 | 44,8 | 49,6 | 21,8 | 31,3 | 44,0 | 69,6 | 63,2 | 2,8 | 8,6 |
| 6 | 2 | 7/13/2016 | 80 | 2,5 | 44,6 | 44,6 | 10,8 | 55,4 | 45,3 | 19,5 | 28,8 | 43,0 | 67,6 | 63,6 | 3,3 | 8,1 |
| 6 | 2 | 7/18/2016 | 40 | 1 | 52,5 | 42,4 | 5,1 | 47,5 | 44,7 | 13,8 | 24,6 | 30,8 | 56,0 | 55,0 | 2,9 | 7,8 |
| 6 | 2 | 8/17/2016 | 75 | 1,5 | 64 | 29,9 | 6,1 | 36 | 35,6 | 13,4 | 20,3 | 37,6 | 65,8 | 57,1 | 2,8 | 9,1 |
| 6 | 2 | 8/31/2016 | 85 | 2,5 | 43,7 | 49,7 | 6,6 | 56,3 | 37,8 | 12,1 | 21,1 | 31,9 | 57,1 | 55,9 | 3,2 | 7,2 |
| 6 | 2 | 9/12/2016 | 85 | 2,5 | | | | | | | | | | | | |
| 6 | 2 | 9/28/2016 | 75 | 2,5 | 38,1 | 41,3 | 20,6 | 61,9 | 59,3 | 29,3 | 40,0 | 49,3 | 73,2 | 67,4 | 2,9 | 9,1 |
| 6 | 2 | 10/10/2016 | 55 | 2,5 | 68,4 | 20 | 11,6 | 31,6 | 64,0 | 41,2 | 48,8 | 64,3 | 84,4 | 76,2 | 3,1 | 9,1 |
| 6 | 2 | 10/24/2016 | 20 | 1 | 80,1 | 15,2 | 4,7 | 19,9 | 45,4 | 14,5 | 24,2 | 31,9 | 59,8 | 53,3 | 1,8 | 4,7 |
| 6 | 2 | 11/7/2016 | 60 | 2 | 71 | 23,8 | 5,2 | 29 | 42,5 | 23,1 | 28,8 | 54,4 | 80,3 | 67,8 | 2,5 | 10,4 |
| 6 | 2 | 11/10/2016 | 30 | 1,5 | 65 | 18,2 | 16,7 | 34,9 | 62,6 | 42,4 | 47,6 | 67,7 | 89,0 | 76,1 | 2,7 | 9,8 |

| | | | | | | | | | | | | | | | | |
|---|---|---|---|---|---|---|---|---|---|---|---|---|---|---|---|---|
| 6 | 2 | 11/14/2016 | 15 | 1 | 90,9 | 6,9 | 2,2 | 9,1 | 43,6 | 17,6 | 23,7 | 40,3 | 74,2 | 54,3 | 1,6 | 6,4 |
| 6 | 3 | 7/18/2016 | 40 | 1 | 73,3 | 24,4 | 2,3 | 26,7 | 26,2 | 8,7 | 15,0 | 33,2 | 58,3 | 57,0 | 2,5 | 8,6 |
| 6 | 3 | 8/17/2016 | 40 | 1,5 | 69,1 | 27,0 | 3,9 | 30,9 | 35,1 | 17,0 | 24,3 | 48,4 | 69,9 | 69,2 | 2,8 | 9,8 |
| 6 | 3 | 8/31/2016 | 40 | 1 | 62,4 | 35,9 | 1,7 | 37,6 | 31,1 | 8,2 | 16,3 | 26,4 | 50,4 | 52,3 | 2,3 | 7,9 |
| 6 | 3 | 9/12/2016 | 30 | 1,5 | 74,8 | 21,3 | 3,9 | 25,2 | 32,1 | 15,9 | 21,3 | 49,5 | 74,6 | 66,4 | 2,6 | 9,1 |
| 6 | 3 | 9/28/2016 | 40 | 1,5 | 68,4 | 25,1 | 6,5 | 31,6 | 36,5 | 18,6 | 24,9 | 50,9 | 74,7 | 68,1 | 2,6 | 9,4 |
| 6 | 3 | 10/10/2016 | 60 | 1,5 | 76,9 | 19,1 | 4,0 | 23,1 | 38,3 | 19,1 | 26,5 | 49,9 | 72,1 | 69,3 | 2,3 | 9,5 |
| 6 | 3 | 10/24/2016 | 30 | 1 | 74,6 | 20,4 | 5,0 | 25,4 | 51,6 | 16,6 | 26,5 | 32,2 | 62,6 | 51,4 | 2,0 | 4,7 |
| 6 | 3 | 11/7/2016 | 15 | 2,5 | 82,1 | 11,1 | 6,8 | 17,9 | 77,7 | 56,3 | 63,3 | 72,5 | 89,0 | 81,4 | 2,1 | 9,6 |
| 6 | 3 | 11/10/2016 | 30 | 1,5 | 84,0 | 12,5 | 3,5 | 16 | 42,2 | 22,4 | 27,4 | 53,1 | 81,8 | 64,9 | 1,7 | 5,1 |
| 6 | 3 | 11/14/2016 | 50 | 1,5 | 91,6 | 5,4 | 3,0 | 8,4 | 46,4 | 19,6 | 28,5 | 42,1 | 68,7 | 61,3 | 2,2 | 6,7 |
| 7 | 1 | 6/20/2016 | 60 | 2,5 | 25,2 | 45 | 29,9 | 74,9 | 62,6 | 37,9 | 47,8 | 60,6 | 79,5 | 76,2 | 3,2 | 8,3 |
| 7 | 1 | 6/27/2016 | 18 | 2 | 79,4 | 19,6 | 0,9 | 20,5 | 24,0 | 10,5 | 14,5 | 43,8 | 72,3 | 60,6 | 1,5 | 7,4 |
| 7 | 1 | 7/4/2016 | | | 19,8 | 35,3 | 44,8 | 80,1 | 72,8 | 43,2 | 53,2 | 59,3 | 81,2 | 73,1 | 3,2 | 8,9 |
| 7 | 1 | 7/13/2016 | 80 | 2,5 | 67,3 | 19,2 | 13,5 | 32,7 | 30,8 | 11,4 | 16,0 | 36,8 | 71,1 | 51,8 | 1,1 | 4,2 |
| 7 | 1 | 7/27/2016 | 75 | 2,5 | 40,7 | 46,2 | 13,2 | 59,4 | 38,8 | 20,0 | 25,1 | 51,5 | 79,6 | 64,8 | 2,6 | 9,6 |
| 7 | 1 | 8/17/2016 | 75 | 4 | 19 | 35,6 | 54,4 | 90 | 88,3 | 63,4 | 73,9 | 71,9 | 85,8 | 83,8 | 3,1 | 9,8 |
| 7 | 1 | 8/31/2016 | 90 | 4 | 18,1 | 24,9 | 57,1 | 82 | 85,4 | 62,7 | 70,9 | 73,5 | 88,4 | 83,1 | 2,7 | 10,2 |
| 7 | 1 | 9/12/2016 | 85 | 2 | 24,8 | 58,6 | 16,6 | 75,2 | 43,4 | 20,6 | 29,0 | 47,4 | 71,0 | 66,8 | 2,7 | 9,0 |
| 7 | 1 | 9/21/2016 | 90 | 4 | 15,3 | 29,5 | 55,2 | 84,7 | 85,4 | 57,9 | 68,3 | 67,8 | 84,8 | 79,9 | 3,0 | 9,1 |
| 7 | 1 | 10/3/2016 | 80 | 3 | 5,9 | 42,2 | 51,9 | 94,1 | 80,1 | 40,9 | 56,5 | 51,1 | 72,4 | 70,6 | 3,2 | 8,4 |
| 7 | 1 | 10/24/2016 | 90 | 3 | 16,4 | 38,6 | 45,1 | 83,7 | 81,5 | 46,5 | 61,5 | 57,1 | 75,6 | 75,5 | 3,2 | 8,4 |
| 7 | 1 | 11/7/2016 | 85 | 2,5 | 25,4 | 43,1 | 31,5 | 74,6 | 55,4 | 31,4 | 39,4 | 56,7 | 79,7 | 71,1 | 2,9 | 9,6 |
| 7 | 1 | 11/14/2016 | 75 | 2 | 29 | 41,1 | 29,9 | 71 | 54,6 | 31,6 | 40,3 | 57,9 | 78,5 | 73,7 | 2,6 | 8,8 |
| 7 | 1 | 11/21/2016 | 70 | 2,5 | 43,2 | 30,8 | 25,9 | 56,7 | 63,0 | 40,8 | 48,3 | 64,7 | 84,4 | 76,7 | 3,1 | 9,0 |
| 7 | 2 | 6/27/2016 | 80 | 2 | 26,3 | 60,7 | 13 | 73,7 | 44,3 | 18,0 | 27,7 | 40,6 | 65,0 | 62,5 | 3,0 | 7,9 |
| 7 | 2 | 7/4/2016 | 75 | 2 | 37,5 | 56,3 | 6,1 | 62,4 | 42,4 | 13,7 | 24,4 | 32,3 | 56,3 | 57,5 | 3,3 | 6,3 |
| 7 | 2 | 7/13/2016 | 85 | 2 | 68,9 | 24,2 | 6,9 | 31,1 | 45,7 | 21,2 | 30,0 | 46,4 | 70,7 | 65,6 | 2,8 | 6,8 |
| 7 | 2 | 7/27/2016 | 60 | 2,5 | 65,8 | 21,8 | 12,3 | 34,1 | 62,2 | 43,5 | 51,3 | 69,9 | 84,9 | 82,4 | 2,4 | 8,4 |
| 7 | 2 | 8/17/2016 | 75 | 2 | 29,5 | 61,3 | 9,2 | 70,5 | 45,4 | 14,2 | 24,8 | 31,2 | 57,3 | 54,5 | 2,8 | 7,5 |
| 7 | 2 | 8/31/2016 | 70 | 2 | 32,6 | 49,8 | 17,7 | 67,5 | 51,3 | 24,3 | 34,3 | 47,4 | 71,0 | 66,9 | 3,4 | 8,7 |
| 7 | 2 | 9/12/2016 | 70 | 1,5 | | | | | | | | | | | | |
| 7 | 2 | 9/21/2016 | 40 | 2,5 | 62,9 | 23,9 | 13,2 | 37,1 | 59,8 | 35,4 | 44,0 | 59,3 | 80,6 | 73,6 | 2,6 | 8,1 |

| | | | | | | | | | | | | | | | | |
|---|---|---|---|---|---|---|---|---|---|---|---|---|---|---|---|---|
| 7 | 2 | 10/3/2016 | 40 | 3 | 86,4 | 10,6 | 3 | 13,6 | 47,9 | 25,6 | 32,5 | 53,4 | 78,8 | 67,8 | 2,4 | 5,7 |
| 7 | 2 | 10/24/2016 | 40 | 1,5 | 81,4 | 15,2 | 3,4 | 18,6 | 46,4 | 20,8 | 30,7 | 44,8 | 67,6 | 66,3 | 2,9 | 9,0 |
| 7 | 2 | 11/7/2016 | 40 | 1,5 | 84,2 | 9,1 | 6,8 | 15,9 | 49,1 | 32,3 | 37,6 | 65,7 | 85,9 | 76,5 | 2,2 | 7,5 |
| 7 | 2 | 11/14/2016 | 45 | 2 | 82,9 | 12,3 | 4,8 | 17,1 | 46,4 | 28,4 | 34,7 | 61,3 | 82,0 | 74,8 | 2,8 | 10,0 |
| 7 | 2 | 11/21/2016 | 35 | 1 | 88,1 | 9,8 | 2,1 | 11,9 | 34,6 | 18,3 | 21,6 | 53,0 | 84,9 | 62,4 | 2,0 | 6,5 |
| 7 | 3 | 7/27/2016 | 30 | 1,5 | 84 | 11,9 | 4,1 | 16 | 49,7 | 30,8 | 35,3 | 62,0 | 87,2 | 71,1 | 2,8 | 9,9 |
| 7 | 3 | 8/17/2016 | 30 | 1 | 66,3 | 30,6 | 3,1 | 33,7 | 30,8 | 11,1 | 17,8 | 36,0 | 62,2 | 57,9 | 2,8 | 8,4 |
| 7 | 3 | 8/31/2016 | 40 | 1,5 | 54,2 | 43,1 | 2,7 | 45,8 | 30,0 | 9,7 | 17,0 | 32,3 | 56,9 | 56,7 | 2,9 | 8,1 |
| 7 | 3 | 9/12/2016 | 35 | 1,5 | | | | | | | | | | | | |
| 7 | 3 | 9/21/2016 | 40 | 2,5 | 62,7 | 31,9 | 5,4 | 37,3 | 35,9 | 16,1 | 23,7 | 45,0 | 68,1 | 66,2 | 3,0 | 8,3 |
| 7 | 3 | 10/3/2016 | 40 | 1,5 | 74,5 | 22,5 | 3,0 | 25,5 | 38,9 | 12,3 | 21,1 | 31,6 | 58,5 | 54,1 | 2,8 | 6,6 |
| 7 | 3 | 10/24/2016 | 35 | 1,5 | 92,1 | 4,8 | 3,1 | 7,9 | 48,1 | 27,2 | 32,7 | 56,5 | 83,1 | 68,0 | 1,8 | 7,2 |
| 7 | 3 | 11/7/2016 | 20 | 1 | 78,2 | 17,9 | 3,9 | 21,8 | 38,3 | 17,6 | 23,4 | 45,8 | 75,1 | 61,0 | 2,3 | 6,4 |
| 7 | 3 | 11/14/2016 | 15 | 1 | 92,2 | 6,1 | 1,7 | 7,8 | 38,1 | 16,7 | 23,9 | 43,7 | 69,7 | 62,8 | 1,6 | 8,2 |
| 7 | 3 | 11/21/2016 | 40 | 1,5 | 73,7 | 19,8 | 6,5 | 26,3 | 39,4 | 23,6 | 27,7 | 59,9 | 85,1 | 70,3 | 2,6 | 9,1 |
| 8 | 1 | 6/20/2016 | 50 | 2,5 | 48 | 33,6 | 18,4 | 52 | 53,2 | 34,4 | 40,4 | 64,6 | 85,1 | 75,9 | 2,9 | 9,9 |
| 8 | 1 | 6/27/2016 | 70 | 3 | 28,1 | 43,4 | 28,5 | 71,9 | 54,3 | 38,7 | 43,9 | 71,2 | 88,1 | 80,8 | 2,6 | 9,1 |
| 8 | 1 | 7/4/2016 | 65 | 2 | 33,3 | 36,9 | 29,7 | 66,6 | 58,6 | 41,9 | 46,7 | 71,4 | 89,6 | 79,7 | 2,9 | 9,1 |
| 8 | 1 | 7/13/2016 | 70 | 2 | 36,4 | 43,8 | 19,8 | 63,6 | 62,5 | 25,9 | 38,6 | 41,5 | 67,2 | 61,8 | 3,5 | 8,9 |
| 8 | 1 | 7/18/2016 | 50 | 1,5 | 34,8 | 49,8 | 15,5 | 65,3 | 40,6 | 21,1 | 27,6 | 52,1 | 76,6 | 68,0 | 2,7 | 9,3 |
| 8 | 1 | 9/7/2016 | 30 | 1,5 | 84,4 | 13,9 | 1,6 | 15,5 | 26,6 | 10,0 | 14,5 | 37,4 | 68,8 | 54,3 | 2,8 | 10,3 |
| 8 | 1 | 9/19/2016 | 75 | 2 | 31,1 | 51 | 17,9 | 68,9 | 52,3 | 24,3 | 34,6 | 46,4 | 70,2 | 66,2 | 3,2 | 8,0 |
| 8 | 1 | 9/21/2016 | 60 | 3 | 28,8 | 27 | 44,1 | 71,1 | 91,0 | 69,6 | 78,2 | 76,5 | 89,0 | 85,9 | 2,9 | 9,7 |
| 8 | 1 | 10/3/2016 | 75 | 1,5 | 19,9 | 41,7 | 38,4 | 80,1 | 64,1 | 37,7 | 49,5 | 58,8 | 76,1 | 77,2 | 2,9 | 8,2 |
| 8 | 1 | 10/17/2016 | 75 | 3 | 59,8 | 19,3 | 20,9 | 40,2 | 71,8 | 56,1 | 61,0 | 78,1 | 91,9 | 85,0 | 2,6 | 9,9 |
| 8 | 1 | 10/26/2016 | 80 | 2 | 27 | 39,2 | 33,7 | 72,9 | 63,6 | 40,2 | 49,4 | 63,3 | 81,4 | 77,8 | 2,9 | 8,6 |
| 8 | 1 | 11/10/2016 | 85 | 3 | 18,5 | 29,3 | 52,2 | 81,5 | 76,2 | 53,2 | 61,8 | 69,9 | 86,1 | 81,2 | 2,7 | 9,1 |
| 8 | 1 | 11/16/2016 | 60 | 2 | 37 | 42,8 | 20,2 | 63 | 51,9 | 27,9 | 36,1 | 53,7 | 77,1 | 69,7 | 3,2 | 8,9 |
| 8 | 1 | 11/21/2016 | 55 | 2,5 | 70,8 | 24,7 | 4,5 | 29,2 | 31,7 | 14,6 | 20,4 | 46,0 | 71,4 | 64,4 | 2,9 | 9,1 |
| 8 | 2 | 6/20/2016 | | | 78,9 | 19,9 | 1,1 | 21 | 20,2 | 9,0 | 13,0 | 44,4 | 69,2 | 64,1 | 2,1 | 5,5 |
| 8 | 2 | 6/27/2016 | 50 | 2 | | | | | | | | | | | | |
| 8 | 2 | 7/4/2016 | 50 | 1 | 79,4 | 17,6 | 3 | 20,6 | 29,7 | 12,6 | 17,1 | 42,4 | 73,6 | 57,6 | 2,3 | 8,6 |
| 8 | 2 | 7/13/2016 | 75 | 1,5 | 71,9 | 21,9 | 6,3 | 28,2 | 34,1 | 17,5 | 22,4 | 51,2 | 78,0 | 65,6 | 2,5 | 10,1 |

| | | | | | | | | | | | | | | | | |
|---|---|---|---|---|---|---|---|---|---|---|---|---|---|---|---|---|
| 8 | 2 | 7/18/2016 | 65 | 1 | 19,2 | 69,1 | 11,8 | 80,9 | 40,6 | 17,0 | 25,7 | 41,8 | 66,0 | 63,3 | 3,1 | 7,8 |
| 8 | 2 | 9/7/2016 | 40 | 1,5 | 53 | 38,6 | 8,3 | 46,9 | 49,4 | 18,2 | 28,0 | 36,9 | 65,0 | 56,8 | 3,7 | 7,4 |
| 8 | 2 | 9/19/2016 | 85 | 2 | 36,3 | 45,7 | 18 | 63,7 | 49,0 | 24,7 | 33,4 | 50,3 | 73,8 | 68,2 | 3,0 | 8,6 |
| 8 | 2 | 9/21/2016 | 60 | 1 | 63,5 | 26,3 | 10,2 | 36,5 | 47,6 | 27,5 | 34,3 | 57,9 | 80,2 | 72,2 | 3,0 | 10,4 |
| 8 | 2 | 10/3/2016 | 70 | 2,5 | 58,2 | 28 | 13,8 | 41,8 | 49,0 | 29,0 | 36,0 | 59,3 | 80,6 | 73,6 | 2,8 | 7,4 |
| 8 | 2 | 10/17/2016 | 30 | 1 | 86 | 11,4 | 2,6 | 14 | 33,0 | 18,7 | 23,2 | 56,5 | 80,6 | 70,1 | 2,6 | 9,1 |
| 8 | 2 | 10/26/2016 | 30 | 2,5 | 91,3 | 6,7 | 2,1 | 8,8 | 43,4 | 34,7 | 38,0 | 79,9 | 91,4 | 87,4 | 0,9 | 5,4 |
| 8 | 2 | 11/10/2016 | 35 | 2 | 70,1 | 19 | 10,9 | 29,9 | 48,2 | 28,5 | 35,9 | 59,1 | 79,5 | 74,4 | 2,8 | 8,7 |
| 8 | 2 | 11/16/2016 | 50 | 2 | 69 | 19 | 12 | 31 | 52,4 | 35,6 | 42,3 | 67,9 | 84,0 | 80,8 | 2,4 | 8,6 |
| 8 | 2 | 11/21/2016 | 40 | 1,5 | 89,9 | 8,7 | 1,4 | 10,1 | 45,9 | 26,7 | 32,0 | 58,3 | 83,6 | 69,7 | 2,3 | 8,2 |
| 8 | 3 | 7/18/2016 | 30 | 1 | 80,6 | 17,8 | 1,7 | 19,5 | 31,0 | 14,4 | 21,4 | 46,3 | 67,0 | 69,1 | 2,6 | 9,1 |
| 8 | 3 | 9/7/2016 | 40 | 2 | 48,9 | 47,5 | 3,6 | 51,1 | 29,9 | 10,5 | 17,5 | 35,1 | 60,0 | 58,4 | 3,1 | 6,7 |
| 8 | 3 | 9/19/2016 | 40 | 1,5 | 58,0 | 35,7 | 6,4 | 42,1 | 39,5 | 15,0 | 23,9 | 38,1 | 62,9 | 60,5 | 3,0 | 6,9 |
| 8 | 3 | 9/21/2016 | 30 | 1 | 68,1 | 26,9 | 5,0 | 31,9 | 36,5 | 17,1 | 23,8 | 46,7 | 71,7 | 65,1 | 2,8 | 8,7 |
| 8 | 3 | 10/3/2016 | 20 | 1,5 | 72,2 | 21,6 | 6,3 | 27,9 | 43,2 | 23,3 | 30,6 | 53,9 | 75,9 | 70,9 | 2,5 | 9,1 |
| 8 | 3 | 10/17/2016 | 20 | 18 | 85,0 | 11,0 | 4,0 | 15 | 44,9 | 25,4 | 31,3 | 56,7 | 81,4 | 69,7 | 2,0 | 8,6 |
| 8 | 3 | 10/26/2016 | 40 | 2,5 | 86,7 | 12,1 | 1,2 | 13,3 | 33,7 | 17,5 | 21,8 | 52,0 | 80,3 | 64,7 | 1,6 | 4,9 |
| 8 | 3 | 11/10/2016 | 30 | 1,5 | 77,0 | 15,0 | 8,0 | 23 | 47,1 | 24,0 | 30,9 | 51,1 | 77,9 | 65,6 | 2,3 | 8,9 |
| 8 | 3 | 11/16/2016 | 40 | 2,5 | 80,1 | 14,5 | 5,5 | 20 | 49,3 | 33,3 | 38,8 | 67,7 | 85,9 | 78,8 | 2,6 | 10,1 |
| 8 | 3 | 11/21/2016 | 30 | 1 | 86,3 | 13 | 0,7 | 13,7 | 28,6 | 14,6 | 20,9 | 51,0 | 69,8 | 73,0 | 1,2 | 6,0 |
| 9 | 1 | 6/20/2016 | 75 | 3,5 | 9,4 | 31,3 | 59,4 | 90,7 | 78,1 | 56,1 | 64,1 | 71,8 | 87,4 | 82,1 | 2,7 | 9,2 |
| 9 | 1 | 6/27/2016 | 75 | 3,5 | 14,8 | 44,5 | 40,6 | 85,1 | 67,0 | 47,6 | 54,3 | 71,0 | 87,6 | 81,1 | 2,5 | 9,4 |
| 9 | 1 | 7/4/2016 | 75 | 2,5 | 57,1 | 39,1 | 3,8 | 42,9 | 27,2 | 13,8 | 17,6 | 51,0 | 78,5 | 64,9 | 2,5 | 8,5 |
| 9 | 1 | 7/13/2016 | 70 | 2,5 | 49,2 | 30,9 | 19,9 | 50,8 | 61,9 | 39,0 | 47,3 | 63,0 | 82,5 | 76,4 | 3,1 | 9,4 |
| 9 | 1 | 7/18/2016 | 80 | 2 | | | | | | | | | | | | |
| 9 | 1 | 8/10/2016 | 80 | 4 | 14,7 | 32,8 | 52,5 | 85,3 | 87,2 | 63,3 | 72,5 | 72,6 | 87,3 | 83,2 | 2,7 | 9,4 |
| 9 | 1 | 9/7/2016 | 80 | 3,5 | 9 | 24,6 | 66,5 | 91,1 | 116,5 | 86,8 | 100,7 | 74,5 | 86,2 | 86,4 | 2,9 | 8,7 |
| 9 | 1 | 9/19/2016 | 90 | 3 | 14,4 | 28,4 | 57,2 | 85,6 | 80,6 | 59,8 | 66,9 | 74,1 | 89,3 | 83,0 | 2,7 | 10,0 |
| 9 | 1 | 9/21/2016 | 70 | 3,5 | 6,1 | 22,1 | 71,8 | 93,9 | 122,1 | 97,4 | 108,9 | 79,7 | 89,4 | 89,2 | 2,8 | 9,0 |
| 9 | 1 | 10/3/2016 | 85 | 3 | 5,7 | 39,7 | 54,6 | 94,3 | 88,8 | 53,1 | 67,6 | 59,8 | 78,6 | 76,1 | 3,2 | 9,0 |
| 9 | 1 | 10/17/2016 | 60 | 2 | 70,5 | 25,1 | 4,3 | 29,4 | 33,0 | 13,2 | 19,5 | 40,1 | 67,8 | 59,2 | 2,7 | 8,8 |
| 9 | 1 | 10/26/2016 | 90 | 3 | 23,4 | 36,6 | 39,9 | 76,5 | 71,7 | 48,6 | 57,0 | 67,8 | 85,2 | 79,5 | 3,1 | 9,5 |
| 9 | 1 | 11/10/2016 | 85 | 2,5 | 7,5 | 28 | 64,5 | 92,5 | 97,1 | 77,6 | 86,0 | 79,9 | 90,2 | 88,5 | 2,6 | 9,9 |

| | | | | | | | | | | | | | | | | |
|---|---|---|---|---|---|---|---|---|---|---|---|---|---|---|---|---|
| 9 | 1 | 11/16/2016 | 40 | 1 | 67,1 | 30,4 | 2,4 | 32,8 | 28,3 | 12,9 | 18,0 | 45,8 | 72,0 | 63,6 | 1,8 | 8,0 |
| 9 | 2 | 6/20/2016 | 75 | 1,5 | 74,5 | 32,2 | 2,3 | 34,5 | 33,0 | 13,3 | 20,2 | 40,5 | 66,0 | 61,3 | 2,6 | 8,2 |
| 9 | 2 | 6/27/2016 | 40 | 1,5 | 82,3 | 17,2 | 0,5 | 17,7 | 21,7 | 6,9 | 11,2 | 31,7 | 61,4 | 51,6 | 1,2 | 3,6 |
| 9 | 2 | 7/4/2016 | 65 | 1,5 | 56 | 33,6 | 10,4 | 44 | 43,6 | 20,9 | 30,2 | 48,0 | 69,2 | 69,3 | 2,7 | 7,6 |
| 9 | 2 | 7/13/2016 | 50 | 1 | 68,6 | 28,4 | 3 | 31,4 | 35,0 | 14,1 | 22,2 | 40,3 | 63,6 | 63,4 | 2,8 | 11,0 |
| 9 | 2 | 7/18/2016 | 80 | 2 | 78,4 | 17,2 | 4,4 | 21,6 | 46,0 | 26,5 | 32,0 | 57,6 | 82,7 | 69,6 | 2,9 | 5,9 |
| 9 | 2 | 8/10/2016 | 50 | 1,5 | 47,9 | 41,3 | 10,8 | 52,1 | 45,5 | 17,3 | 27,1 | 38,0 | 63,9 | 59,6 | 2,9 | 8,3 |
| 9 | 2 | 9/7/2016 | 75 | 1,5 | 64,2 | 32,5 | 3,3 | 35,8 | 32,2 | 11,1 | 18,1 | 34,5 | 61,2 | 56,3 | 3,0 | 7,1 |
| 9 | 2 | 9/19/2016 | 50 | 1,5 | 60,2 | 26,4 | 13,4 | 39,8 | 47,7 | 28,7 | 34,6 | 60,1 | 82,9 | 72,5 | 2,6 | 10,4 |
| 9 | 2 | 9/21/2016 | 30 | 1 | 57,8 | 33,4 | 8,7 | 42,1 | 35,6 | 18,9 | 24,7 | 53,1 | 76,5 | 69,4 | 2,5 | 8,3 |
| 9 | 2 | 10/3/2016 | 70 | 2 | 67,4 | 26,9 | 5,8 | 32,7 | 40,8 | 21,5 | 26,8 | 52,6 | 80,1 | 65,7 | 3,1 | 7,2 |
| 9 | 2 | 10/17/2016 | 30 | 1 | 87,7 | 10,8 | 1,4 | 12,2 | 28,9 | 9,4 | 14,1 | 32,5 | 66,4 | 49,0 | 2,6 | 9,1 |
| 9 | 2 | 10/26/2016 | 45 | 2 | 76,3 | 18,2 | 5,6 | 23,8 | 45,5 | 26,0 | 32,1 | 57,2 | 81,1 | 70,6 | 2,5 | 9,2 |
| 9 | 2 | 11/10/2016 | 45 | 2,5 | 52,3 | 23,6 | 24,1 | 47,7 | 68,5 | 49,0 | 54,4 | 71,5 | 90,1 | 79,4 | 2,2 | 10,6 |
| 9 | 2 | 11/16/2016 | 10 | 2 | 92,2 | 5 | 2,8 | 7,8 | 87,6 | 76,3 | 79,2 | 87,1 | 96,4 | 90,4 | 1,7 | 7,2 |
| 9 | 3 | 7/18/2016 | 40 | 2 | 78,4 | 17,2 | 4,4 | 21,6 | 46,0 | 26,5 | 32,0 | 57,6 | 82,7 | 69,6 | 2,9 | 5,9 |
| 9 | 3 | 8/10/2016 | 40 | 1,5 | 46,0 | 46,2 | 7,8 | 54 | 37,9 | 13,7 | 21,4 | 36,2 | 64,2 | 56,5 | 3,1 | 7,5 |
| 9 | 3 | 9/7/2016 | 50 | 1,5 | 38,1 | 51,6 | 10,3 | 61,9 | 39,6 | 16,7 | 25,2 | 42,1 | 66,2 | 63,6 | 3,0 | 7,2 |
| 9 | 3 | 9/19/2016 | 40 | 1 | 61,2 | 31,6 | 7,2 | 38,8 | 37,1 | 19,1 | 25,0 | 51,3 | 76,3 | 67,3 | 2,6 | 8,6 |
| 9 | 3 | 9/21/2016 | 60 | 2,5 | 13,7 | 70,5 | 15,8 | 86,3 | 49,0 | 20,1 | 31,4 | 41,0 | 64,0 | 64,1 | 3,2 | 7,6 |
| 9 | 3 | 10/3/2016 | 30 | 2 | 83,5 | 14,1 | 2,5 | 16,6 | 41,2 | 23,6 | 29,6 | 57,2 | 79,7 | 71,8 | 2,9 | 8,4 |
| 9 | 3 | 10/17/2016 | 25 | 1,5 | 69,7 | 21,9 | 8,4 | 30,3 | 50,2 | 27,4 | 34,7 | 54,7 | 79,1 | 69,1 | 2,8 | 9,5 |
| 9 | 3 | 10/26/2016 | 30 | 2 | 90,2 | 7,3 | 2,6 | 9,9 | 43,4 | 29,5 | 33,8 | 68,1 | 87,4 | 77,9 | 1,4 | 5,0 |
| 9 | 3 | 11/10/2016 | 10 | 1 | 87,7 | 8,3 | 4,0 | 12,3 | 52,9 | 29,0 | 34,9 | 54,8 | 83,1 | 65,9 | 1,2 | 5,8 |
| 9 | 3 | 11/16/2016 | 20 | 1,5 | 87 | 10,8 | 2,2 | 13 | 36,4 | 16,9 | 21,8 | 46,6 | 77,7 | 59,9 | 1,7 | 6,8 |
| 10 | 1 | 6/20/2016 | 60 | 4 | 28,5 | 39,5 | 32 | 71,5 | 77,6 | 46,7 | 59,9 | 60,1 | 78,0 | 77,1 | 3,1 | 8,3 |
| 10 | 1 | 6/27/2016 | 20 | 1,5 | 83,3 | 16,5 | 0,2 | 16,7 | 21,9 | 8,3 | 12,1 | 37,7 | 68,0 | 55,3 | 1,5 | 2,5 |
| 10 | 1 | 7/4/2016 | | | 14,7 | 34,6 | 50,6 | 85,2 | 95,3 | 63,0 | 77,0 | 66,1 | 81,8 | 80,8 | 3,3 | 8,9 |
| 10 | 1 | 7/13/2016 | 90 | 3 | 43 | 35,9 | 21,1 | 57 | 60,3 | 37,7 | 45,7 | 62,6 | 82,5 | 75,9 | 3,0 | 8,4 |
| 10 | 1 | 7/27/2016 | 50 | | 47,6 | 38,6 | 13,9 | 52,5 | 41,6 | 23,4 | 29,4 | 56,2 | 79,4 | 70,8 | 2,7 | 9,6 |
| 10 | 1 | 8/17/2016 | 85 | 4 | 16,6 | 31,1 | 52,3 | 83,4 | 107,0 | 66,8 | 85,0 | 62,5 | 78,7 | 79,4 | 3,4 | 9,1 |
| 10 | 1 | 8/31/2016 | 90 | 2 | 26,4 | 31,4 | 42,2 | 73,6 | 90,4 | 58,8 | 72,1 | 65,1 | 81,6 | 79,7 | 3,2 | 9,2 |
| 10 | 1 | 9/12/2016 | 85 | 2 | 26 | 51,2 | 22,8 | 74 | 58,9 | 30,6 | 41,5 | 51,9 | 73,6 | 70,5 | 3,3 | 9,1 |

| | | | | | | | | | | | | | | | | |
|---|---|---|---|---|---|---|---|---|---|---|---|---|---|---|---|---|
| 10 | 1 | 9/28/2016 | 80 | 2,5 | 18,1 | 56,7 | 25,2 | 81,9 | 57,8 | 30,3 | 41,5 | 52,4 | 72,9 | 71,9 | 3,1 | 7,8 |
| 10 | 1 | 10/10/2016 | 80 | 2,5 | 41,8 | 33,1 | 25,1 | 58,2 | 57,4 | 35,1 | 43,4 | 61,1 | 80,8 | 75,6 | 2,8 | 8,8 |
| 10 | 1 | 10/24/2016 | 70 | 1,5 | 56,4 | 31 | 12,5 | 43,5 | 45,5 | 20,0 | 28,1 | 44,0 | 71,1 | 61,8 | 2,8 | 8,7 |
| 10 | 1 | 11/7/2016 | 85 | 3 | 19,5 | 33,6 | 46,9 | 80,5 | 88,4 | 59,6 | 71,7 | 67,4 | 83,2 | 81,0 | 2,7 | 9,8 |
| 10 | 1 | 11/14/2016 | 90 | 3,5 | 16,9 | 30,4 | 52,6 | 83 | 80,6 | 53,3 | 63,6 | 66,1 | 83,7 | 78,9 | 3,1 | 9,7 |
| 10 | 1 | 11/21/2016 | 90 | 3 | 8,6 | 34,8 | 56,5 | 91,3 | 96,9 | 64,1 | 77,6 | 66,2 | 82,6 | 80,1 | 3,5 | 9,0 |
| 10 | 2 | 6/27/2016 | 60 | 2 | 74,3 | 23,7 | 2 | 25,7 | 25,1 | 10,4 | 15,6 | 41,5 | 67,0 | 61,9 | 2,6 | 11,0 |
| 10 | 2 | 7/4/2016 | 80 | 2 | 60,9 | 36,5 | 2,5 | 39 | 34,9 | 10,6 | 18,6 | 30,4 | 56,8 | 53,4 | 2,7 | 9,2 |
| 10 | 2 | 7/13/2016 | 85 | 2,5 | 55,3 | 36,2 | 8,5 | 44,7 | 45,3 | 17,8 | 27,7 | 39,2 | 64,1 | 61,1 | 3,0 | 8,1 |
| 10 | 2 | 7/27/2016 | 65 | 2,5 | 60,3 | 26,8 | 12,9 | 39,7 | 56,3 | 37,7 | 43,9 | 67,0 | 85,9 | 78,0 | 2,9 | 10,0 |
| 10 | 2 | 8/17/2016 | 75 | 2 | 43,3 | 43,7 | 13 | 56,7 | 48,0 | 20,8 | 31,2 | 43,3 | 66,5 | 65,1 | 3,0 | 8,0 |
| 10 | 2 | 8/31/2016 | 30 | 1 | 83,4 | 16 | 0,7 | 16,7 | 24,0 | 7,8 | 13,1 | 32,4 | 59,2 | 54,8 | 2,5 | 10,5 |
| 10 | 2 | 9/12/2016 | 75 | 1 | | | | | | | | | | | | |
| 10 | 2 | 9/28/2016 | 80 | 2 | 29,9 | 44,4 | 25,8 | 70,2 | 58,5 | 31,5 | 41,5 | 53,8 | 75,8 | 71,0 | 2,9 | 8,4 |
| 10 | 2 | 10/10/2016 | 50 | 3 | 63,8 | 19,6 | 16,6 | 36,2 | 66,9 | 45,6 | 53,8 | 68,1 | 84,7 | 80,4 | 2,9 | 8,3 |
| 10 | 2 | 10/24/2016 | 45 | 2 | 72,2 | 22,6 | 5,2 | 27,8 | 40,2 | 20,6 | 27,0 | 51,2 | 76,4 | 67,0 | 2,6 | 7,4 |
| 10 | 2 | 11/7/2016 | 40 | 2,5 | 82 | 9,5 | 8,5 | 18 | 61,6 | 45,7 | 49,1 | 74,2 | 93,1 | 79,7 | 2,4 | 10,7 |
| 10 | 2 | 11/14/2016 | 30 | 2,5 | 68 | 23,2 | 8,9 | 32,1 | 54,0 | 31,7 | 40,0 | 58,7 | 79,2 | 74,1 | 2,9 | 10,0 |
| 10 | 2 | 11/21/2016 | 60 | 2,5 | 64,3 | 21,3 | 14,3 | 35,6 | 63,0 | 40,3 | 49,3 | 64,0 | 81,7 | 78,4 | 2,5 | 9,5 |
| 10 | 3 | 7/27/2016 | 45 | 2,5 | 79,4 | 13,4 | 7,2 | 20,6 | 56,9 | 38,5 | 45,4 | 67,6 | 84,7 | 79,8 | 2,6 | 10,1 |
| 10 | 3 | 8/17/2016 | 60 | 1 | 69,1 | 15,5 | 15,5 | 31 | 66,8 | 44,7 | 52,0 | 66,9 | 86,0 | 77,7 | 2,6 | 9,0 |
| 10 | 3 | 8/31/2016 | 30 | 1 | 72,0 | 26,6 | 1,4 | 28 | 26,6 | 9,2 | 15,6 | 34,7 | 59,1 | 58,7 | 2,2 | 9,1 |
| 10 | 3 | 9/12/2016 | 30 | 1 | 80,2 | 14,4 | 5,3 | 19,7 | 47,7 | 27,6 | 34,5 | 57,9 | 79,9 | 72,4 | 2,8 | 8,2 |
| 10 | 3 | 9/28/2016 | 40 | 1 | 41,3 | 45,7 | 13 | 58,7 | 38,1 | 19,5 | 26,1 | 51,3 | 74,7 | 68,6 | 2,6 | 8,4 |
| 10 | 3 | 10/10/2016 | 40 | 2 | 59,8 | 29,3 | 10,9 | 40,2 | 53,4 | 30,7 | 38,0 | 57,5 | 80,7 | 71,3 | 2,8 | 6,6 |
| 10 | 3 | 10/24/2016 | 35 | 1,5 | 80,2 | 15,3 | 4,5 | 19,8 | 51,3 | 24,9 | 35,0 | 48,6 | 71,2 | 68,3 | 2,4 | 9,0 |
| 10 | 3 | 11/7/2016 | 15 | 2 | 58,6 | 25,7 | 15,7 | 41,4 | 50,7 | 30,8 | 37,2 | 60,6 | 82,7 | 73,3 | 2,2 | 9,3 |
| 10 | 3 | 11/14/2016 | 20 | 1 | 85,2 | 12,4 | 2,3 | 14,7 | 35,7 | 15,5 | 22,0 | 43,4 | 70,3 | 61,7 | 1,6 | 7,1 |
| 10 | 3 | 11/21/2016 | 45 | 2,5 | 83,7 | 14 | 2,3 | 16,3 | 41,5 | 16,5 | 25,8 | 39,8 | 63,9 | 62,2 | 1,5 | 5,0 |
| 11 | 1 | 6/20/2016 | 80 | 3 | 9,3 | 42,9 | 47,8 | 90,7 | 70,7 | 44,9 | 54,2 | 63,5 | 82,9 | 76,6 | 3,0 | 9,3 |
| 11 | 1 | 6/27/2016 | 60 | 2,5 | 28,2 | 44,2 | 27,6 | 71,8 | 54,4 | 32,3 | 39,4 | 59,4 | 82,0 | 72,4 | 2,7 | 9,3 |
| 11 | 1 | 7/4/2016 | 90 | 4 | 41,9 | 47,5 | 10,6 | 58,1 | 39,4 | 17,1 | 24,2 | 43,3 | 70,6 | 61,4 | 3,0 | 10,0 |
| 11 | 1 | 7/13/2016 | 85 | 2,5 | 16,1 | 35,2 | 48,7 | 83,9 | 83,8 | 47,7 | 62,6 | 56,9 | 76,1 | 74,7 | 3,3 | 8,2 |

| | | | | | | | | | | | | | | | | |
|---|---|---|---|---|---|---|---|---|---|---|---|---|---|---|---|---|
| 11 | 1 | 7/18/2016 | 80 | 2 | 78,9 | 20,4 | 0,7 | 21,1 | 28,3 | 11,5 | 17,1 | 40,7 | 67,3 | 60,5 | 2,8 | 10,0 |
| 11 | 1 | 8/10/2016 | 85 | 3 | 13,6 | 53,4 | 33 | 86,4 | 65,8 | 32,2 | 45,6 | 48,9 | 70,7 | 69,2 | 3,1 | 8,0 |
| 11 | 1 | 9/7/2016 | 60 | 2 | 14,2 | 33,5 | 52,3 | 85,8 | 76,7 | 45,4 | 57,4 | 59,1 | 79,0 | 74,8 | 3,0 | 8,6 |
| 11 | 1 | 9/19/2016 | 85 | 2,5 | 34,5 | 60,5 | 5 | 65,5 | 34,6 | 12,9 | 21,3 | 37,2 | 60,3 | 61,6 | 3,1 | 7,8 |
| 11 | 1 | 9/21/2016 | 50 | 2 | 40,7 | 47,4 | 11,9 | 59,3 | 36,0 | 18,0 | 24,3 | 50,2 | 74,1 | 67,7 | 2,6 | 9,9 |
| 11 | 1 | 10/3/2016 | 70 | 2,5 | 31,6 | 50,2 | 18,2 | 68,4 | 47,1 | 23,3 | 32,3 | 49,5 | 72,3 | 68,5 | 3,1 | 8,3 |
| 11 | 1 | 10/17/2016 | 85 | 2 | 35,8 | 38,9 | 25,3 | 64,2 | 54,9 | 32,3 | 40,3 | 58,9 | 80,3 | 73,4 | 2,9 | 9,9 |
| 11 | 1 | 10/26/2016 | 80 | 2 | 24 | 54,1 | 22 | 76,1 | 50,6 | 23,4 | 34,3 | 46,3 | 68,3 | 67,8 | 2,9 | 7,6 |
| 11 | 1 | 11/7/2016 | 85 | 3 | 18,3 | 43,2 | 38,5 | 81,7 | 71,0 | 40,6 | 52,3 | 57,2 | 77,6 | 73,7 | 3,2 | 8,9 |
| 11 | 2 | 6/20/2016 | 65 | 1,5 | 80,4 | 18,2 | 1,4 | 19,6 | 23,7 | 9,8 | 14,3 | 41,5 | 68,7 | 60,4 | 2,4 | 9,5 |
| 11 | 2 | 6/27/2016 | 50 | 1,5 | 69,4 | 26,9 | 3,7 | 30,6 | 28,2 | 12,3 | 17,9 | 43,6 | 68,8 | 63,4 | 2,3 | 7,1 |
| 11 | 2 | 7/4/2016 | 60 | 1,5 | 79,4 | 18,8 | 1,7 | 20,5 | 27,8 | 10,8 | 17,0 | 38,8 | 63,7 | 60,9 | 2,7 | 9,1 |
| 11 | 2 | 7/13/2016 | 85 | 2,5 | 43,5 | 37 | 19,6 | 56,6 | 58,6 | 33,9 | 44,0 | 57,8 | 77,0 | 75,1 | 2,8 | 9,0 |
| 11 | 2 | 7/18/2016 | 65 | 1 | 60 | 38,3 | 1,8 | 40,1 | 31,6 | 8,6 | 16,9 | 27,1 | 50,9 | 53,3 | 2,8 | 9,5 |
| 11 | 2 | 8/10/2016 | 60 | 2 | 39,6 | 46,1 | 14,2 | 60,3 | 48,8 | 20,8 | 31,0 | 42,7 | 67,3 | 63,5 | 3,0 | 8,6 |
| 11 | 2 | 9/7/2016 | 90 | 2 | 38,4 | 52,1 | 9,5 | 61,6 | 40,7 | 15,0 | 24,2 | 36,8 | 61,8 | 59,5 | 3,2 | 7,8 |
| 11 | 2 | 9/19/2016 | 80 | 2 | 28,7 | 50,3 | 21 | 71,3 | 51,9 | 26,1 | 36,5 | 50,2 | 71,4 | 70,4 | 3,0 | 8,5 |
| 11 | 2 | 9/21/2016 | 75 | 1,5 | 41,7 | 50,6 | 7,6 | 58,2 | 39,2 | 14,9 | 23,8 | 38,0 | 62,6 | 60,7 | 2,8 | 8,2 |
| 11 | 2 | 10/3/2016 | 70 | 3 | 61,9 | 23,4 | 14,7 | 38,1 | 54,3 | 36,9 | 42,5 | 68,0 | 86,8 | 78,3 | 2,8 | 8,0 |
| 11 | 2 | 10/17/2016 | 20 | 1 | 84,1 | 14,4 | 1,4 | 15,8 | 31,2 | 11,5 | 17,6 | 36,8 | 65,3 | 56,3 | 1,5 | 5,5 |
| 11 | 2 | 10/26/2016 | 50 | 1,5 | 32,3 | 54,3 | 13,4 | 67,7 | 46,5 | 19,1 | 30,6 | 41,0 | 62,3 | 65,9 | 3,1 | 7,1 |
| 11 | 2 | 11/7/2016 | 35 | 1 | 83 | 12,6 | 4,3 | 16,9 | 55,1 | 32,7 | 41,2 | 59,4 | 79,4 | 74,8 | 2,7 | 9,7 |
| 11 | 3 | 7/13/2016 | 45 | 1 | 44 | 50,4 | 5,5 | 55,9 | 32,9 | 12,7 | 20,1 | 38,5 | 62,8 | 61,2 | 2,9 | 8,1 |
| 11 | 3 | 7/18/2016 | 40 | 2 | 76,1 | 18,9 | 5 | 23,9 | 38,4 | 20,4 | 26,6 | 52,9 | 76,4 | 69,3 | 1,9 | 6,7 |
| 11 | 3 | 8/10/2016 | 35 | 1 | 62,3 | 33,9 | 3,8 | 37,7 | 33,1 | 12,2 | 20,5 | 37,0 | 59,9 | 61,8 | 2,4 | 7,8 |
| 11 | 3 | 9/7/2016 | 40 | 2 | 58,3 | 32,7 | 9,1 | 41,8 | 35,4 | 17,6 | 24,0 | 49,8 | 73,3 | 67,9 | 2,7 | 8,3 |
| 11 | 3 | 9/19/2016 | 60 | 2 | 42,1 | 46,8 | 11,1 | 57,9 | 41,1 | 17,4 | 27,0 | 42,4 | 64,6 | 65,6 | 2,9 | 7,8 |
| 11 | 3 | 9/21/2016 | 60 | 2,5 | 57,8 | 39,3 | 2,9 | 42,2 | 26,9 | 10,1 | 16,6 | 37,5 | 60,8 | 61,6 | 2,9 | 8,4 |
| 11 | 3 | 10/3/2016 | 40 | 1 | 68,6 | 22,4 | 9,0 | 31,4 | 56,1 | 32,8 | 41,9 | 58,4 | 78,3 | 74,6 | 2,7 | 8,7 |
| 11 | 3 | 10/17/2016 | 40 | 2,5 | 76,1 | 19,3 | 4,6 | 23,9 | 38,6 | 18,3 | 25,3 | 47,5 | 72,4 | 65,6 | 2,1 | 7,4 |
| 11 | 3 | 10/26/2016 | 35 | 2 | 82,0 | 14,7 | 3,4 | 18,1 | 49,9 | 34,1 | 40,5 | 68,4 | 84,3 | 81,1 | 2,1 | 7,1 |
| 11 | 3 | 11/7/2016 | 35 | 2 | 88,6 | 10,0 | 1,4 | 11,4 | 43,3 | 23,8 | 30,0 | 54,9 | 79,2 | 69,4 | 1,4 | 6,1 |
| 12 | 1 | 6/20/2016 | 70 | 3 | 29,9 | 53,5 | 16,6 | 70,1 | 48,0 | 22,8 | 32,7 | 47,5 | 69,7 | 68,0 | 2,9 | 8,5 |

| | | | | | | | | | | | | | | | | |
|---|---|---|---|---|---|---|---|---|---|---|---|---|---|---|---|---|
| 12 | 1 | 6/27/2016 | 40 | 2 | 57,9 | 39,6 | 2,6 | 42,2 | 26,5 | 12,3 | 17,2 | 46,7 | 71,8 | 64,9 | 2,7 | 10,0 |
| 12 | 1 | 7/4/2016 | 45 | 2 | 69 | 29 | 2 | 31 | 27,3 | 14,4 | 19,5 | 52,9 | 74,1 | 71,4 | 2,2 | 11,9 |
| 12 | 1 | 7/13/2016 | 75 | 2,5 | 38,5 | 22,1 | 39,4 | 61,5 | 76,8 | 49,8 | 58,1 | 64,9 | 85,8 | 75,7 | 3,4 | 9,6 |
| 12 | 1 | 7/27/2016 | 80 | 2,5 | 17,9 | 47,2 | 34,9 | 82,1 | 85,0 | 38,4 | 56,2 | 45,2 | 68,4 | 66,2 | 3,5 | 8,6 |
| 12 | 1 | 8/17/2016 | 80 | 2 | 27,8 | 46,7 | 25,5 | 72,2 | 50,7 | 27,2 | 35,8 | 53,5 | 75,8 | 70,7 | 2,9 | 8,7 |
| 12 | 1 | 8/31/2016 | 85 | 3,5 | 26,7 | 18,7 | 54,6 | 73,3 | 95,4 | 71,9 | 80,6 | 75,4 | 89,3 | 84,5 | 2,9 | 9,7 |
| 12 | 1 | 9/12/2016 | 70 | 1,5 | 35,9 | 55,3 | 8,8 | 64,1 | 36,8 | 14,8 | 23,2 | 40,2 | 63,8 | 63,0 | 3,0 | 8,1 |
| 12 | 1 | 9/28/2016 | 70 | 2,5 | 30,8 | 50,9 | 18,4 | 69,3 | 49,8 | 23,5 | 32,9 | 47,2 | 71,5 | 66,1 | 3,1 | 8,2 |
| 12 | 1 | 10/10/2016 | 70 | 2,5 | 44,5 | 31,1 | 24,4 | 55,5 | 61,0 | 40,6 | 47,9 | 66,6 | 84,8 | 78,5 | 3,2 | 9,2 |
| 12 | 1 | 10/24/2016 | 80 | 2 | 31 | 34,8 | 34,1 | 68,9 | 66,6 | 35,0 | 45,0 | 52,5 | 77,8 | 67,5 | 3,1 | 8,9 |
| 12 | 1 | 11/7/2016 | 90 | 3 | 12,2 | 32,4 | 55,4 | 87,8 | 85,3 | 59,4 | 69,3 | 69,7 | 85,7 | 81,3 | 3,1 | 10,0 |
| 12 | 1 | 11/14/2016 | 75 | 2 | 15,5 | 48,2 | 36,3 | 84,5 | 71,7 | 35,5 | 46,7 | 49,5 | 76,1 | 65,1 | 3,2 | 10,3 |
| 12 | 1 | 11/21/2016 | 80 | 3,5 | 33,6 | 36 | 30,4 | 66,4 | 62,6 | 38,8 | 50,2 | 62,0 | 77,3 | 80,2 | 2,7 | 7,9 |
| 12 | 2 | 6/20/2016 | | | 69,9 | 25,1 | 5 | 30,1 | 33,5 | 15,6 | 20,8 | 46,4 | 74,9 | 62,0 | 2,9 | 9,7 |
| 12 | 2 | 6/27/2016 | 80 | 2 | 35,9 | 59,3 | 4,8 | 64,1 | 37,6 | 13,0 | 22,3 | 34,6 | 58,2 | 59,4 | 3,3 | 6,7 |
| 12 | 2 | 7/4/2016 | 60 | 1 | 79,5 | 19,2 | 1,3 | 20,5 | 28,6 | 10,1 | 16,5 | 35,2 | 61,0 | 57,7 | 2,9 | 8,2 |
| 12 | 2 | 7/13/2016 | 85 | 2,5 | 29,4 | 62,8 | 7,8 | 70,6 | 43,1 | 13,8 | 24,2 | 32,0 | 57,0 | 56,3 | 3,2 | 7,1 |
| 12 | 2 | 7/27/2016 | 65 | 2,5 | | | | | | | | | | | | |
| 12 | 2 | 8/17/2016 | 40 | 1 | 83,3 | 16,7 | 0 | 16,7 | 25,4 | 6,2 | 13,9 | 24,4 | 44,6 | 54,8 | 0,0 | 0,0 |
| 12 | 2 | 8/31/2016 | 75 | 1,5 | 43,2 | 49,8 | 7 | 56,8 | 44,5 | 14,2 | 24,9 | 32,0 | 57,1 | 56,0 | 3,2 | 7,5 |
| 12 | 2 | 9/12/2016 | 40 | 1 | | | | | | | | | | | | |
| 12 | 2 | 9/28/2016 | 50 | 1,5 | | | | | | | | | | | | |
| 12 | 2 | 10/10/2016 | 65 | 2 | 89,7 | 8,1 | 2,2 | 10,3 | 41,2 | 18,9 | 25,5 | 46,0 | 74,2 | 62,0 | 1,5 | 4,1 |
| 12 | 2 | 10/24/2016 | 35 | 1 | 82,5 | 12,9 | 4,6 | 17,5 | 48,8 | 23,0 | 30,1 | 47,2 | 76,4 | 61,8 | 3,3 | 8,7 |
| 12 | 2 | 11/7/2016 | 45 | 1,5 | 67,7 | 19,2 | 13,2 | 32,4 | 53,2 | 36,6 | 42,3 | 68,8 | 86,5 | 79,5 | 2,4 | 9,4 |
| 12 | 2 | 11/14/2016 | 40 | 2 | 71 | 19,9 | 9 | 28,9 | 50,5 | 35,3 | 41,8 | 69,9 | 84,3 | 82,9 | 2,2 | 9,6 |
| 12 | 2 | 11/21/2016 | 40 | 2 | 30 | 42,1 | 27,9 | 70 | 60,9 | 35,7 | 46,9 | 58,7 | 76,2 | 77,1 | 2,8 | 7,6 |
| 12 | 3 | 7/27/2016 | 60 | 1,5 | 79,5 | 14,4 | 6,1 | 20,5 | 48,9 | 27,1 | 34,0 | 55,3 | 79,6 | 69,5 | 3,0 | 7,9 |
| 12 | 3 | 8/17/2016 | 30 | 1 | 42,6 | 50,0 | 7,5 | 57,5 | 35,5 | 14,7 | 23,0 | 41,5 | 64,0 | 64,9 | 2,7 | 7,3 |
| 12 | 3 | 8/31/2016 | 30 | 1 | 82,7 | 14,7 | 2,6 | 17,3 | 37,7 | 16,4 | 24,3 | 43,5 | 67,5 | 64,5 | 2,7 | 9,7 |
| 12 | 3 | 9/12/2016 | 60 | 1,5 | | | | | | | | | | | | |
| 12 | 3 | 9/28/2016 | 45 | 1,5 | 68,4 | 25,1 | 6,5 | 31,6 | 36,5 | 18,6 | 24,9 | 50,9 | 74,7 | 68,1 | 2,6 | 9,4 |
| 12 | 3 | 10/10/2016 | 60 | 2,5 | 70,7 | 18,9 | 10,4 | 29,3 | 66,3 | 43,0 | 52,7 | 64,8 | 81,5 | 79,5 | 3,2 | 9,1 |

| | | | | | | | | | | | | | | | | |
|---|---|---|---|---|---|---|---|---|---|---|---|---|---|---|---|---|
| 12 | 3 | 10/24/2016 | 45 | 2 | 74,7 | 20,9 | 4,4 | 25,3 | 41,1 | 21,2 | 26,7 | 51,6 | 79,2 | 65,1 | 2,2 | 6,4 |
| 12 | 3 | 11/7/2016 | 10 | 1 | 83,6 | 9,9 | 6,5 | 16,4 | 76,2 | 55,2 | 62,0 | 72,4 | 89,1 | 81,3 | 2,1 | 9,6 |
| 12 | 3 | 11/14/2010 | 40 | 2,5 | 89,6 | 8,4 | 2,0 | 10,4 | 30,9 | 11,3 | 15,7 | 36,7 | 72,1 | 50,8 | 1,2 | 4,4 |
| 12 | 3 | 11/21/2016 | 35 | 2 | 86,6 | 9,5 | 3,9 | 13,4 | 61,3 | 39,9 | 47,2 | 65,1 | 84,5 | 77,0 | 2,0 | 8,0 |
