# Supplementary material for: Seminal plasma amino acid profile in different breeds of chicken: Role of seminal plasma on sperm cryoresistance
Source: PLoS One. 2019 Jan 4;14(1):e0209910. doi: 10.1371/journal.pone.0209910 (PMC6319765; doi:10.1371/journal.pone.0209910)
Supplement: S5 Dataset — (PDF) [file pone.0209910.s005.pdf]

S5 Dataset. Tunel + of sperm of 12 Spanish rooster breeds (fresh, frozen without plasma and frozen with plasma).

Year 2016 w/o/p= without plasma w/p= with plasma

Month	Day	Rooster Breed	TUNEL + (%)		
			Fresh	w/p	w/o/p
September	19	Birchen Leonesa	13,5		18
September	21	Birchen Leonesa	32		19
October	3	Birchen Leonesa	17,8	25	18
October	17	Birchen Leonesa	15		22
October	26	Birchen Leonesa	2	25	32
November	16	Birchen Leonesa	18,4	33	21
November	21	Birchen Leonesa	15	22	10
November	22	Birchen Leonesa	12	25	22
June	20	Black Castellana		30	
June	27	Black Castellana		44	
July	4	Black Castellana		21	
August	17	Black Castellana			5
August	31	Black Castellana		10	12
September	10	Black Castellana	1,8		23
September	26	Black Castellana	7,2		
October	24	Black Castellana		45	20
November	7	Black Castellana		22	21
November	14	Black Castellana	11,2	20	13
November	16	Black Castellana	5,3	23	5
November	22	Black Castellana	7	20	15
July	11	Black-Red Andaluza		20	
July	18	Black-Red Andaluza		30	
September	7	Black-Red Andaluza			15
September	10	Black-Red Andaluza	8,2		
September	19	Black-Red Andaluza	5	17	10
September	26	Black-Red Andaluza	6,6	17	13
October	10	Black-Red Andaluza		20	7
October	17	Black-Red Andaluza	6	55	14
October	26	Black-Red Andaluza	3,5	35	20
November	10	Black-Red Andaluza			17
November	16	Black-Red Andaluza	2	18	5
June	27	Black-Barred Andaluza		25	
August	17	Black-Barred Andaluza			7
August	31	Black-Barred Andaluza			25
September	10	Black-Barred Andaluza	21		10
September	21	Black-Barred Andaluza	6,8		16
October	3	Black-Barred Andaluza	3,9	32	13
October	24	Black-Barred Andaluza		18	33
November	14	Black-Barred Andaluza	12,6	25	22
November	21	Black-Barred Andaluza	11	32	40
November	22	Black-Barred Andaluza	14	21	15
June	20	Blue Andaluza		15	
July	4	Blue Andaluza		27	
July	11	Blue Andaluza		23	

July	13	Blue Andaluza			12
September	7	Blue Andaluza		38	15
September	10	Blue Andaluza	7,5		
September	19	Blue Andaluza	4	15	5
September	21	Blue Andaluza		12	
September	26	Blue Andaluza	10		
October	17	Blue Andaluza	3,5		
October	26	Blue Andaluza	3	19	6
November	16	Blue Andaluza	15	36	7
July	4	Buff Prat		26	
July	11	Buff Prat		10	
August	17	Buff Prat		31	
August	31	Buff Prat			12
September	10	Buff Prat	13,9		
September	26	Buff Prat	5	31	18
October	10	Buff Prat			22
October	24	Buff Prat		27	10
November	7	Buff Prat		27	
November	14	Buff Prat	12	27	15
November	21	Buff Prat	9	13	10
November	22	Buff Prat	8	31	20
June	27	Quail Castellana		16	
July	4	Quail Castellana		10	
July	18	Quail Castellana		12	
September	7	Quail Castellana		13	
September	19	Quail Castellana	11,6		
September	21	Quail Castellana	9,4	9	13
October	3	Quail Castellana	8,4	10	10
October	17	Quail Castellana	9,75	28	
October	26	Quail Castellana	2		
November	10	Quail Castellana		23	5
November	16	Quail Castellana	10,8	25	15
July	11	Quail Silver Castellana		18	
July	13	Quail Silver Castellana			13
August	17	Quail Silver Castellana			12
August	31	Quail Silver Castellana		26	
September	10	Quail Silver Castellana	2,3		18
September	26	Quail Silver Castellana	4,3	18	
October	10	Quail Silver Castellana		30	
October	24	Quail Silver Castellana			15
November	7	Quail Silver Castellana		21	16
November	10	Quail Silver Castellana		20	12
November	14	Quail Silver Castellana	13,4	25	17
July	11	Red Villafranquina		40	
September	10	Red Villafranquina	8,5		9
September	26	Red Villafranquina	9,8	17	15
October	24	Red Villafranquina		46	15
October	26	Red Villafranquina			10
November	7	Red Villafranquina		21	10
November	14	Red Villafranquina	11	26	20

November	21	Red Villafranquina	10	49	
November	22	Red Villafranquina	10	24	21
July	4	Red-Barred Vasca		21	
September	19	Red-Barred Vasca	3,8	25	25
September	21	Red-Barred Vasca	19	18	9
October	3	Red-Barred Vasca	7,2		16
October	17	Red-Barred Vasca	3,4	13	
October	26	Red-Barred Vasca	2,9	45	20
November	7	Red-Barred Vasca		33	
June	27	White-Faced Spanish		33	
August	17	White-Faced Spanish		40	
August	31	White-Faced Spanish			28
September	7	White-Faced Spanish			18
September	10	White-Faced Spanish	42		22
September	21	White-Faced Spanish	24	35	
October	3	White-Faced Spanish	16	25	23
November	7	White-Faced Spanish		14	
November	14	White-Faced Spanish	16	40	30
November	21	White-Faced Spanish	22	25	15
November	22	White-Faced Spanish	18	35	22
June	20	White Prat		20	
August	10	White Prat			15
August	17	White Prat		50	
September	19	White Prat	12,2		
September	21	White Prat	1,5	22	12
October	3	White Prat	7,6	15	
October	17	White Prat	2	17	14
October	26	White Prat	3	22	
November	10	White Prat		18	
November	16	White Prat	24	26	10
